# Long COVID or Post-acute Sequelae of COVID-19 (PASC): An Overview of Biological Factors That May Contribute to Persistent Symptoms

**DOI:** 10.3389/fmicb.2021.698169

**Published:** 2021-06-23

**Authors:** Amy D. Proal, Michael B. VanElzakker

**Affiliations:** ^1^PolyBio Research Foundation, Kenmore, WA, United States; ^2^Division of Neurotherapeutics, Massachusetts General Hospital, Harvard Medical School, Boston, MA, United States

**Keywords:** COVID-19, long COVID, SARS-CoV-2, microbiome, vagus, brainstem, infection, virus

## Abstract

The novel virus severe acute respiratory syndrome coronavirus 2 (SARS-CoV-2) has caused a pandemic of coronavirus disease 2019 (COVID-19). Across the globe, a subset of patients who sustain an acute SARS-CoV-2 infection are developing a wide range of persistent symptoms that do not resolve over the course of many months. These patients are being given the diagnosis Long COVID or Post-acute sequelae of COVID-19 (PASC). It is likely that individual patients with a PASC diagnosis have different underlying biological factors driving their symptoms, none of which are mutually exclusive. This paper details mechanisms by which RNA viruses beyond just SARS-CoV-2 have be connected to long-term health consequences. It also reviews literature on acute COVID-19 and other virus-initiated chronic syndromes such as post-Ebola syndrome or myalgic encephalomyelitis/chronic fatigue syndrome (ME/CFS) to discuss different scenarios for PASC symptom development. Potential contributors to PASC symptoms include consequences from acute SARS-CoV-2 injury to one or multiple organs, persistent reservoirs of SARS-CoV-2 in certain tissues, re-activation of neurotrophic pathogens such as herpesviruses under conditions of COVID-19 immune dysregulation, SARS-CoV-2 interactions with host microbiome/virome communities, clotting/coagulation issues, dysfunctional brainstem/vagus nerve signaling, ongoing activity of primed immune cells, and autoimmunity due to molecular mimicry between pathogen and host proteins. The individualized nature of PASC symptoms suggests that different therapeutic approaches may be required to best manage care for specific patients with the diagnosis.

## Introduction

**FIGURE 1:**
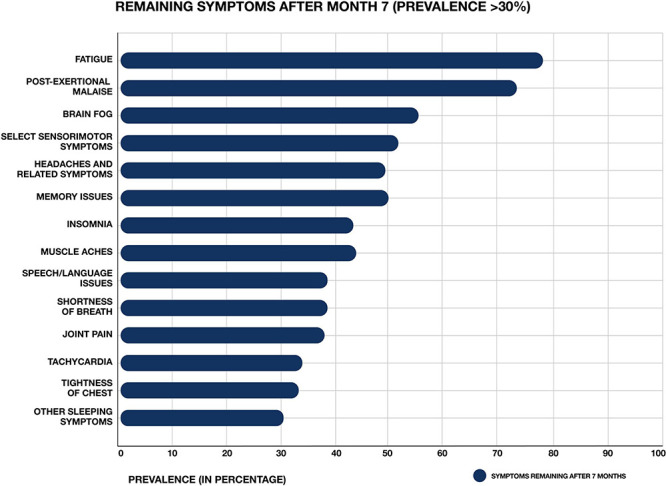
Most common symptoms remaining after 7 months in 966 respondents from a cohort of suspected and confirmed COVID-19 cases. Results obtained via an international web-based survey. Image adapted with permission from Davis et al. (2020).

The novel virus severe acute respiratory syndrome coronavirus 2 (SARS-CoV-2) has resulted in a global pandemic of coronavirus disease 2019 (COVID-19) (Hiscott et al., 2020). Classic cases of acute COVID-19 are characterized by respiratory symptoms, fever, and gastrointestinal problems (Larsen et al., 2020). However, patients can present with a wide range of other symptoms, including neurological issues suggesting central nervous system (CNS) involvement (Harapan and Yoo, 2021). Acute COVID-19 cases range in length and severity. Many patients are asymptomatic, while others require hospitalization and ventilation (Cunningham et al., 2021). Overall, an average case of COVID-19 lasts between 1 and 4 weeks. However, across the globe, a subset of patients who sustain an acute SARS CoV-2 infection are developing a wide range of persistent symptoms that do not resolve over the course of many months (Carfì et al., 2020; Davis et al., 2020; Huang C. et al., 2021) (Figure 1). One study of COVID-19 patients who were followed for up to 9 months after illness found that approximately 30% reported persistent symptoms (Logue et al., 2021). These patients are being given the diagnosis Long COVID, post-acute COVID-19 syndrome (PACS), or post-acute sequelae of COVID-19 (PASC).

Post-acute sequelae of COVID-19 is being diagnosed in patients who developed severe acute COVID-19, but also in patients who experienced only mild or asymptomatic cases. For example, Tabacof et al. (2020) reported a range of long-term symptoms in a cohort of previously confirmed or presumed COVID-19 patients whose acute symptoms were largely managed without the need for hospitalization. Another team documented persistent COVID-19 symptoms in 1,407 subjects with confirmed SARS-CoV-2 infection (Huang Y. et al., 2021). Symptoms included fatigue and muscle weakness, insomnia, palpitations, chronic rhinitis, dysgeusia, chills, sore throat, and headache. 27% of subjects reported persistent symptoms after 60 days, with patients aged 50 ± 20 years comprising 72% of cases. Women were more likely to report persistent symptoms, and ∼32% of subjects reporting symptoms at 61+ days after infection were asymptomatic at the time of initial SARS-CoV-2 testing.

While the development of long-term symptoms following SARS-CoV-2 infection is sometimes framed as novel or mysterious, it is actually an expected phenomenon. Most well-studied viral or bacterial pathogens have been connected to the development of chronic symptoms in a subset of infected patients (Bannister, 1988; Rebman and Aucott, 2020; Komaroff and Bateman, 2021). For example, Ebola virus is associated with a chronic syndrome that can develop after acute infection (Wilson et al., 2018), with reservoirs of Ebola sometimes identified in “anatomical sanctuaries” in patient tissue months or years after the virus has cleared from the blood (Lo et al., 2017; Keita et al., 2019).

Some PASC patients meet the diagnostic criteria for myalgic encephalomyelitis/chronic fatigue syndrome (ME/CFS) – a neuroinflammation-linked condition characterized by a range of debilitating chronic symptoms including severe fatigue, musculoskeletal pain, and post-exertional malaise (worsening of symptoms following exertion) (Carruthers et al., 2011; Clayton, 2015; Kedor et al., 2021; Komaroff and Bateman, 2021). Overlap between the PASC and ME/CFS diagnoses is not surprising, since most cases of ME/CFS begin with a viral infection, or involve multiple exposures to viral and bacterial pathogens over time (Rasa et al., 2018).

Pathogens most commonly implicated in ME/CFS development include neurotrophic herpesviruses and enteroviruses. Several studies have found that active HHV-6 infection is more common in ME/CFS than controls (Komaroff, 2006). Enteroviruses, several species of which can be acquired via respiratory infection, have been identified in brain, skeletal muscle, and stomach biopsy specimens of certain patients with ME/CFS (Gow et al., 1991; McGarry et al., 1994; Richardson, 2001; Chia and Chia, 2008). Other respiratory pathogens have also been linked to the development of ME/CFS-like symptoms (Magnus et al., 2015). For example, one team studied 233 SARS survivors approximately 4 years after initial infection, and found that 27.1% met the modified 1994 Centers for Disease Control and Prevention (CDC) criteria for chronic fatigue syndrome (Lam et al., 2009).

It is likely that the different pathogens implicated in ME/CFS development are capable of dysregulating host gene expression, immunity, and metabolism via similar mechanisms, leading to similar sets of chronic symptoms in ME/CFS-diagnosed patients (VanElzakker, 2013; Proal and Marshall, 2018). However, a range of additional biological factors, including changes in host microbiome composition and activity, can also contribute to ME/CFS development (Giloteaux et al., 2016; Newberry et al., 2018). The same trend may be true of PASC cases, in which SARS-CoV-2 infection may instigate or exacerbate different biological abnormalities in patients with the diagnosis.

This paper reviews relevant ME/CFS, neuroscience, microbiology, and single-stranded RNA virus literatures to explore a range of biological factors that may contribute to the development of chronic symptoms after acute COVID-19, none of which are mutually exclusive. These include consequences from acute injury caused by SARS-CoV-2, persistent reservoirs of SARS-CoV-2 in certain tissues, re-activation of other neurotrophic pathogens under conditions of COVID-19 immune dysregulation, SARS-CoV-2 interactions with host microbiome/virome communities, clotting/coagulation issues, disrupted brainstem/vagus nerve signaling, ongoing activity of primed immune cells, and autoimmunity due to molecular mimicry between pathogen and host peptides.

## Acute COVID-19

In order to best understand persistent symptoms arising from SARS-CoV-2 infection we must first review major trends associated with SARS CoV-2 activity in patients with acute COVID-19. SARS-CoV-2 is a positive-sense single-stranded RNA virus (V’kovski et al., 2021). It is one of seven coronaviruses capable of infecting humans (Corman et al., 2018). Compared with other coronaviruses (e.g., HCoV-NL63, HCoV-229E, and HCoV-OC43) that are pathogenic to humans but generally drive only mild clinical symptoms, SARS-CoV-2 more closely resembles MERS-CoV or SARS-CoV (sometimes called SARS-CoV-1) in that it is capable of causing severe disease (Zhu et al., 2020).

Many COVID-19 patients are asymptomatic (∼40–45%) (Oran and Topol, 2020) or exhibit mild to moderate symptoms (Zheng P. et al., 2020). However, approximately 15% progress to severe pneumonia, with ∼5% eventually developing acute respiratory distress syndrome (ARDS), septic shock and/or multiple organ failure (Cao, 2020; Huang et al., 2020). The most common symptoms of acute COVID-19 are fever, fatigue, diarrhea, and respiratory symptoms such as cough, sore throat and shortness of breath (Larsen et al., 2020). However, some patients develop neurological manifestations ranging from mild symptoms such as anosmia, dizziness, and headache to more severe cerebrovascular disease, seizures, encephalitis, or the Guillain–Barré syndrome (Berlit et al., 2020). Other extrapulmonary manifestations of COVID-19 include acute kidney injury, hyperglycemia, thrombotic complications, myocardial dysfunction and arrhythmia, acute coronary syndromes, and hepatocellular injury (Gupta et al., 2020; Robba et al., 2020).

These diverse COVID-19 symptoms partially reflect the fact that SARS-CoV-2 can infect a wide range of human cell types. The spike subunit of SARS CoV-2 binds the human angiotensin-converting enzyme 2 (ACE2) receptor to infect and enter host cells (Hoffmann et al., 2020). Viral cell entry additionally requires priming of the spike protein by cellular serine proteases such as TMPRSS2 and TMPRSS4. ACE2 is expressed along the entire human respiratory system and in brain endothelium and vascular smooth muscle cells (Hamming et al., 2004). Single-cell RNA-sequencing studies have also confirmed expression of ACE2 and TMPRSS2 in a wide range of cell types including esophageal keratinocytes, renal proximal tubules, pancreatic β-cells, and gastrointestinal epithelial cells (Gupta et al., 2020; Puelles et al., 2020; Qi et al., 2020).

Recent work has further clarified CNS cellular expression of ACE2 and other genes that may contribute to COVID-19. For example, Matschke et al. (2020) performed an *in silico* analysis of publicly available datasets to determine which CNS cell types might be prone to SARS-CoV-2 infection. They analyzed genes that can contribute to viral entry into the cell and viral persistence, including ACE2, TMPRSS2, TMPRSS4, TPCN2, CTSL, and NRP1. They found that these genes are expressed in neurons, glial cells, and endothelial cells, suggesting their possible capacity to support SARS-CoV-2 infection.

Like all pathogens, SARS-CoV-2 employs a number of mechanisms to disable and evade the host immune response (Lucas et al., 2001; Bowie and Unterholzner, 2008; Taefehshokr et al., 2020). These include the ability to replicate within double-membrane vesicles that are not detected by host pathogen pattern recognition receptors (Taefehshokr et al., 2020). SARS-CoV-2 also dysregulates the host interferon response (Ribero et al., 2020). Interferons are cytokines secreted by host cells in response to viral infection. They bind to cell surface receptors and act as transcription factors, regulating the expression of hundreds of genes whose protein products target viruses at many levels (Acharya et al., 2020). SARS-CoV-2 expresses at least 10 proteins that allow it to either counteract the induction or escape the antiviral activity of interferons (Ribero et al., 2020), allowing the virus to better survive by rendering the host innate immune response inefficient.

Despite this innate immune disruption, SARS-CoV-2 can initiate host immune signaling pathways. If the virus is not successfully contained, this results in the production of proinflammatory cytokines such as interlukin-6, and the recruitment of neutrophils and myeloid cells (Gubernatorova et al., 2020). This leads to hyperinflammation, and in some cases, a cytokine storm syndrome (Chen and Quach, 2021). Severe COVID-19 can also result in functional exhaustion and decreased numbers of T lymphocytes, (particularly CD4+ T cells, CD8+ T cells) and natural killer cells (Diao et al., 2020; Zheng M. et al., 2020). Impaired T cell responses can result from deficient interferon production driven by SARS-CoV-2, as interferons promote the survival and effector functions of T cells.

SARS-CoV-2 can also drive multi-organ injury via stimulation of clotting cascades (Pretorius et al., 2020a) and related thromboinflammation, dysregulation of the renin–angiotensin–aldosterone system, and endothelial cell damage (Grobler et al., 2020; Gupta et al., 2020). Infection-mediated endothelial injury and endothelialitis (marked by the presence of activated macrophages and neutrophils) can trigger excessive thrombin production, inhibit fibrinolysis, and activate complement pathways in a manner that leads to microvascular dysfunction and microthrombi deposition.

## The Neuroinvasive and Neurotrophic Potential of SARS-CoV-2

Autopsy, animal, and organoid model studies show that, like SARS-CoV, SARS-CoV-2 is able to reach and infect cells of the CNS, infect neurons, and produce neuroinflammation (Matschke et al., 2020; Song et al., 2020; Song et al., 2021). Indeed, SARS-CoV-2 may be capable of transport up and down nerves and neuronal axons (Lima et al., 2020; Rangon et al., 2020; Song et al., 2020; Karuppan et al., 2021).

One pathway by which SARS-CoV-2 may reach the CNS is via hematogenous spread from heavily infected airways and lungs. Systemic inflammation that increases blood brain barrier (BBB) permeability would facilitate this kind of spread. The circumventricular organs are brain structures with fenestrated capillaries and high permeability. This normally allows circulating but non-BBB-crossing mediators to directly affect brain function. However, during acute infection this permeability can also allow for pathogen neuroinvasion. This can occur directly or via a “Trojan horse” mechanism in which host immune cells infected with intracellular pathogens are actively transported into the CNS (Dando et al., 2014; Lauer et al., 2018). Perhaps importantly, several circumventricular organs have relatively high ACE2 expression (Doobay et al., 2007) likely due to the fact that angiotensin II is a peptide hormone that does not easily cross the BBB.

Like other circumventricular organs, the choroid plexus lacks a tight junction BBB at the endothelium but contains tight junction epithelial cells forming the blood-cerebrospinal fluid (CSF) barrier. This layer produces CSF and serves an immune function, partly by facilitating the exchange of nutrients, waste, and peripheral immune cells between the bloodstream and CSF (Thompson et al., 2020). Some pathogens have evolved to enter the brain from blood by exploiting this polarized choroid plexus epithelium (Lauer et al., 2018). In a human-pluripotent-stem-cell-derived organoid model, SARS-CoV-2 exhibited tropism for choroid plexus basal (vascular side) epithelium, despite more abundant ACE2 on the apical (CSF) side (Pellegrini et al., 2020). Furthermore, SARS-CoV-2 caused epithelium damage and barrier leakage, which could facilitate neuroinflammation by allowing increased entry of circulating cytokines and immune cells – including cells infected in a Trojan horse fashion – into the CNS.

SARS-CoV-2 may also enter the central nervous system via the nasal cavity, through the foramina of the cribriform plate, and into the olfactory epithelium. There, the virus can exploit the close vicinity of olfactory sensory neurons whose axons project into the olfactory bulb of the brain (Montalvan et al., 2020). Olfactory entry of SARS-CoV-2 into the CNS is now supported by multiple studies. Meinhardt et al. (2021) analyzed the olfactory mucosa, its nervous projections, and several CNS regions in 33 individuals who died from COVID-19. SARS-CoV-2 RNA and/or protein were identified in anatomically distinct regions of both the nasopharynx and brain, including the medulla oblongata of the brainstem (Meinhardt et al., 2021). SARS-CoV-2 RNA levels were highest within the olfactory mucosa sampled directly under the cribriform plate (*n* = 20 of 30).

Other autopsy studies have identified SARS-CoV-2 RNA or protein in the brainstem of humans and animals (de Melo et al., 2020). Matschke et al. (2020) identified SARS-CoV-2 RNA or protein in 21 of 40 (53%) of COVID-19 autopsied brains, with both SARS-CoV-2 RNA and protein detected in 8 of 40 (20%) of brains (Matschke et al., 2020). Immunohistochemistry demonstrated SARS-CoV-2 viral proteins in vagal and glossopharyngeal cranial nerves originating from the lower brainstem and in isolated cells of the brainstem’s medulla oblongata. This is consistent with the brainstem’s relatively high expression of ACE2 (Lukiw et al., 2020). The brainstem is also the site of the area postrema circumventricular organ, itself a site of ACE2 expression (Doobay et al., 2007). Matschke et al. (2020) additionally reported diffuse activation of microglia and infiltration of cytotoxic T lymphocytes in brainstem and cerebellum tissue.

A smaller study of 18 COVID-19 autopsies focused on microvascular changes in the olfactory bulb and brainstem. They found infiltrating macrophages and activated astrocytes and microglia in the perivascular spaces of 13 brains. *Ex vivo* 11.7 Tesla high-resolution magnetic resonance imaging found punctate hyperintensities in 9 of 13 patients’ brain tissue, representing areas of microvascular injury and fibrinogen leakage. No evidence of SARS-CoV-2 RNA was reported after testing a selection of brain structures from a subset of samples (for example, the medulla of 1 subject and the olfactory bulb of 3 subjects) (Lee et al., 2021, Supplementary Table 5).

With these acute COVID-19 trends in mind, we will now discuss different biological factors that may contribute to the development of persistent symptoms in patients with a PASC diagnosis.

## SARS-CoV-2 Can Cause Injury to One or Multiple Organs or Tissues

Long-term symptoms in some PASC patients may be due to consequences from organ or tissue injury caused by SARS-CoV-2, or associated clotting or inflammatory processes during acute COVID-19 (Del Rio et al., 2020). For example, Guler et al. (2021) found that 4 months after SARS-CoV-2 infection, severe COVID-19 was associated with significant radiological and functional abnormalities indicative of lung parenchymal and small airway disease. Another early analysis of COVID-19 patients upon hospital discharge found that more than a third of recovered patients develop lung fibrotic abnormalities (Rai et al., 2020). Pulmonary fibrosis is characterized by scarring of the lungs. The condition can present as stable in response to injury or infection, or can become progressive (marked by periods of rapid exacerbation) (McDonald, 2021). Both stable and progressive fibrotic lung disease result in excessive deposition of extracellular matrix molecules such as fibronectin, collagen, and laminin in parenchymal lung tissue. This leads to epithelial/endothelial injury and thickened alveolar walls, which can hinder gas exchange in the lungs and increase symptoms of fatigue, dyspnea, and exercise intolerance.

SARS-CoV-2 can also drive acute kidney injury (Robbins-Juarez et al., 2020) in a manner that may contribute to PASC symptoms. For example, Huang C. et al. (2021) found that 6 months after acute COVID-19, 35% of patients displayed a decreased estimated glomerular filtration rate (eGFR). Infection with SARS-CoV-2 is also associated with the development of pediatric multisystem inflammatory syndrome (MIS-C), manifestations of which can cause severe organ inflammation and dysfunction in children, with over 10% of cases showing acute kidney injury (Belay et al., 2021). More information on MIS-C and other SARS-CoV-2 organ injury-related sequelae can be found in the excellent review from Nalbandian et al. (2021). However, in some cases, long-term decreased eGFR and MIS-C were not present during acute COVID-19. For example, 13% of subjects in the Huang C. et al. (2021) study developed new-onset reduction of eGFR after displaying normal renal function during acute COVID-19. One retrospective study found that 810 of 1,075 COVID-19-associated MIS-C cases were asymptomatic during their COVID-19 illness (Belay et al., 2021). This suggests that biological factors beyond organ injury alone may contribute to chronic symptoms in such patients.

## SARS-CoV-2 Appears Capable of Persistence in Certain Tissues

It is also possible that, at least in some PASC patients, SARS-CoV-2 may drive chronic symptoms by persisting in certain body sites or tissue reservoirs after acute infection. A growing number of studies show that some patients infected with SARS-CoV-2 do not successfully clear the virus over long periods of time (Liotti et al., 2020; Sun et al., 2020; Vibholm et al., 2021). In such studies, confirmation of SARS-CoV-2 in patient samples is generally assessed via identification of viral RNA and/or proteins. While identification of SARS-CoV-2 RNA could technically represent “inert” RNA, the possibility is unlikely because inert RNA in the human body is rapidly degraded (Houseley and Tollervey, 2009; Fabre et al., 2014). Nearly every human cell type, and human tears, saliva, mucus, perspiration, and extracellular spaces express RNAase enzymes that rapidly degrade inert RNA (Sorrentino, 2010; Gupta et al., 2013). Indeed, overcoming RNAse activity was a central challenge in mRNA vaccine development (Pardi et al., 2018). It follows that if inert SARS-CoV-2 RNA and/or RNA fragments drive symptoms in some PASC patients, the molecular mechanisms of inert RNA persistence must be better delineated.

Immune responses can also be used to infer the possible persistence of SARS-CoV-2 in certain patients. In a study of 203 patients, Vibholm et al. (2021) found that after 90 days, 5.3% of subjects remained positive for SARS-CoV-2 via RT-PCR nasopharyngeal testing (Plebani, 2021). While there were no differences in SARS-CoV-2 antibody levels between the PCR positive and negative subjects, the PCR positive group displayed SARS-CoV-2-specific CD8 T-cell responses of significantly increased breadth and magnitude, leading the team to suggest that such subjects might still harbor replicating virus.

Gaebler et al. (2021) studied COVID-19 antibody responses in a larger cohort of patients assessed at 1.3 and 6.2 months after SARS-CoV-2 infection. They found that while IgM and IgG anti-spike protein receptor binding domain (RBD) antibody titers decreased significantly, levels of RBD-specific memory B cells remained unchanged. This indicated possible continued antibody evolution, potentially due to small amounts of SARS-CoV-2 antigen or lack of complete viral clearance. To test this possibility, they obtained intestinal biopsy samples from certain study subjects. They identified SARS-CoV-2 RNA and protein in 7 of 14 of the biopsy samples obtained from asymptomatic COVID-19 patients with negative nasal-swab PCR, at an average of 4 months after acute infection.

Another team found SARS-CoV-2 RNA in olfactory mucosa samples collected from 4 patients with negative nasopharyngeal swab SARS-CoV-2 RNA tests but ongoing loss of smell after acute COVID-19 disease (samples were collected 110–196 days after COVID-19 onset) (de Melo et al., 2020). RT-qPCR revealed high levels of SARS-CoV-2 RNA in 3 of the olfactory tissue mucosa samples, and immunostaining revealed SARS-CoV-2 protein antigens in 3 samples.

Immunosuppression may facilitate SARS-CoV-2 persistence (Lancman et al., 2020; Kemp et al., 2021; Tehrani et al., 2021). One team documented SARS-CoV-2 persistence in an antiphospholipid syndrome patient who was administered a wide range of immunosuppressive drugs over 154 days (Choi et al., 2020). Viral infectivity studies confirmed infectious SARS-CoV-2 virus in nasopharyngeal samples obtained from the patient at days 75 and 143 of COVID-19. Examination of the patient’s tissue after death showed the highest SARS-CoV-2 RNA levels in the spleen and lungs. Phylogenetic analysis was consistent with persistent infection and accelerated viral evolution. SARS-CoV-2 mutated in the patient over time to evolve new amino acid changes in its spike protein and receptor binding domain.

A separate report found that late-stage SARS-CoV-2 S variants isolated from the same patient contained mutations that conferred resistance to a common class of SARS-CoV-2 neutralizing antibodies isolated from a healthy COVID-19 convalescent donor (Clark et al., 2021). Resistance extended to monoclonal antibodies used clinically, and to polyclonal serum immunoglobulins obtained from four healthy convalescent donors. This suggests that in some patients, SARS-CoV-2 may be able to evade the immune response in a manner that allows it to drive persistent symptoms. Indeed, some recent COVID-19 variants have spike protein mutations that allow for increased immune evasion, including resistance to neutralizing antibodies or escape from HLA-restricted cellular immunity (McCallum et al., 2021; Motozono et al., 2021; Planas et al., 2021). These mutations could potentially facilitate SARS-CoV-2 persistence in non-immunocompromised individuals.

Persistence of SARS-CoV-2 in some patients with PASC symptoms is not unexpected. The literature is replete with examples of single-stranded RNA virus persistence, spanning decades of research on samples obtained from living human patients, autopsy studies, and animal studies (Viola et al., 1978; Riddell et al., 2007; Doi et al., 2016; Randall and Griffin, 2017; Ireland et al., 2020). Persistence of single-stranded RNA viruses in the central nervous system has been documented on multiple occasions. In a 1986 paper on the topic, Kristensson and Norrby explain that “Although it would seem difficult for RNA viruses to persist in the brain under conditions of normal immune defense mechanisms, representatives of at least seven of the established families of RNA viruses have been shown capable of causing persistent infections under these conditions” (Kristensson and Norrby, 1986).

Single-stranded RNA viruses appear to employ a range of mechanisms to establish persistent infections (Randall and Griffin, 2017). For example, hepatitis C virus (HCV) may be able to hijack cellular factors, such as microRNAs that bind its genome, to shield it from degradation by the 5′–3′ exoribonuclease Xrn1 (Li et al., 2013). This prevents the innate immune response from recognizing and regulating HCV transcription, replication, and genomic RNA abundance. Modifications (insertions and deletions) at the 5′ and/or 3′ ends of the genomes of other single-stranded RNA viruses, such as coxsackieviruses and hantaviruses, may also allow such pathogens to establish persistent infections (Meyer and Southern, 1997; Meyer and Schmaljohn, 2000; Kim et al., 2005).

Dozens of studies show coronaviruses capable of persistence, with some coronaviruses tied to chronic disease development (Arbour et al., 1999; Chan et al., 2004). One study found that in primates infected with two different coronaviruses, the viruses persisted, replicated, and disseminated in the central nervous system, leading to demyelination in the brain (Murray et al., 1992b). Coronavirus RNA and/or antigen have also been found in human multiple sclerosis (MS) brains examined at autopsy, including in both plaque and non-plaque areas of brainstem, cortex, and spinal cord samples (Murray et al., 1992a).

Other respiratory viruses have been found in patient tissue long after acute infection. For example, Castro et al. (2020) identified infectious influenza virus A (IAV) in tonsil tissue removed from children with persistently enlarged tonsils who did not have active respiratory infections at the time of sample collection. Immune cells such as B and T CD8^+^ lymphocytes in examined tonsil tissue also contained IAV antigens. The team concluded that, “Taken together, these results suggest that human lymphoid tissues can be sites of silent IAV infections with possible impact on virus shedding to the population” (Castro et al., 2020).

Some enteroviruses, including those that drive respiratory symptoms, also appear capable of persistence and have been connected to chronic disease (Zhang et al., 2013; Genoni et al., 2017). Enteroviruses of the family picornaviridae include the coxsackieviruses, poliovirus, echoviruses, and rhinoviruses (Baggen et al., 2018). These single-stranded RNA viruses cause about 10–15 million infections each year in the United States alone. Several members of the enterovirus family including polioviruses types 1–3 and enterovirus 71/D68 are neurotrophic and able to drive severe CNS infections (Tang and Holmes, 2017).

**FIGURE 2:**
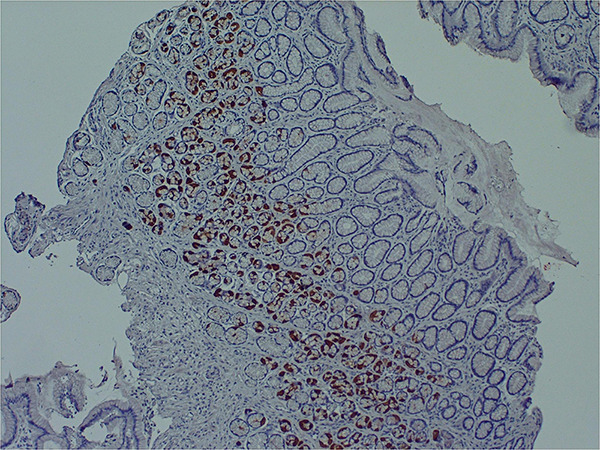
Enteroviral capsid protein 1 in the stomach biopsy of an ME/CFS patient by immunoperoxidase staining (100×) (Chia et al., 2015). This type of chronic viral infection is difficult to identify without the use of special techniques such as antibody staining or nucleic acid amplification of biopsies taken from symptomatic areas, since viral cultures are rarely positive. Original image courtesy of Dr. John Chia.

Several research teams have found enteroviruses in the blood and pancreas of patients with type 1 diabetes, and enteroviruses have been associated with increased risk of type 1 diabetes in prospective studies (Tauriainen et al., 2011; Oikarinen et al., 2012). A number of teams have identified enteroviruses and their proteins in tissue samples obtained from patients with ME/CFS or ME/CFS-like symptoms (Yousef et al., 1988; Dowsett et al., 1990; Chia et al., 2010; Chia et al., 2015). For example, Chia and Chia found enterovirus VP1 protein and RNA in stomach biopsy specimens obtained from 165 ME/CFS patients with chronic abdominal complaints. 82% of ME/CFS specimens stained positive for enterovirus VP1 protein, compared to 20% of control specimens (Chia and Chia, 2008) (Figure 2). A non-cytolytic form of enteroviral infection was cultured from 5 ME/CFS specimens. Positive staining was found in repeat stomach biopsy specimens taken from 6 ME/CFS patients at the onset of symptoms and again 2–8 years later.

Enteroviruses have also been found in ME/CFS brain and muscle tissue (Cunningham et al., 1991; Gow et al., 1991; Richardson, 2001). For example, McGarry et al. (1994) examined the central nervous system of a woman with ME/CFS who died by suicide for the presence of enteroviruses. Positive PCR sequences with similarity to coxsackievirus B3 were identified in brain samples from the hypothalamus and brainstem, and also in muscle and heart samples. No enteroviral sequences were identified in any tissue obtained from four control subjects.

Multiple research teams have identified Zika virus (ZIKV) RNA in men’s semen more than 6 months after initial infection (Nicastri et al., 2016; Huits et al., 2017; Mead et al., 2018). Other studies, including those in primates, strongly support the possibility that, in some people exposed to ZIKV, low-levels of the single-stranded RNA virus may persist in a range of tissues and nerves (Aid et al., 2017). For example, in rhesus macaques infected with ZIKV, Hirsch et al. (2017) found ZIKV RNA in secondary lymphoid tissues, peripheral nervous tissue, joints, and organs of the female reproductive tract up to 35 days post-infection (the end of the study period).

Many Ebola survivors also endure a range of chronic symptoms — termed post-Ebola syndrome — that include musculoskeletal pain, fatigue, headaches, and severe vision problems (Wilson et al., 2018). In one large study of Ebola survivors, 9.8% of 277 male subjects tested positive for Ebola RNA in at least one semen sample (Keita et al., 2019). The probability of remaining positive for Ebola viral RNA in semen was 93.02% after 3 months and 60.12% after 6 months. One breast milk sample was positive for Ebola RNA at day 58, and another positive for Ebola RNA 500 days after hospital discharge. Ebola RNA identified in such body sites appears, as least in some cases, to be viable virus. Indeed, a recent Ebola case in Guinea was linked to transmission following latent Ebola infection of between 5 and 7 years since an original 2014–2016 Makona strain outbreak, rather than new Ebola virus spillover (Virological, 2021).

Antibody responses in Ebola survivors also suggest that Ebola RNA identified in post-Ebola syndrome patients may indicate the presence of low levels of persistent viable virus. Adaken et al. (2021) found that that levels of neutralizing and total antibodies continue to fluctuate in the plasma of a high proportion of Ebola survivors. The authors contend that this periodic antibody resurgence might follow periods of low-grade Ebola virus replication in body sites/tissues shielded from a full-blown immune response. These include the eyes, central nervous system and testes – so-called ‘immunologically privileged sites’ where tolerance to antigens could mitigate inflammation and tissue damage, but might allow Ebola itself to persist over long periods of time.

Animal model studies that track the activity of viral RNA over time also suggest that the RNA identified in post-Ebola syndrome and related syndromes may not be inert. For example, in a mouse model of measles virus (MV) neuronal infection, Miller et al. (2019) showed that MV RNA remains detectable in the neurons/CNS of mice long after initial infection, and in the absence of overt disease. Under such conditions, viral replication is suppressed by the adaptive immune response. However, when the team experimentally induced depletion of adaptive immune cells associated with a loss of T resident memory lymphocytes within the brain, viral transcription and translation recurred. This correlated with development of a new CNS disease in the mice characterized by severe gait and motor problems that was distinct from their acute infectious symptoms. The team concluded that their results demonstrate that “what were once considered ‘resolved’ RNA viral infections may, in fact, induce diseases later in life that are distinct from those caused by acute infection.”

There is no firm consensus on whether patients harboring SARS-CoV-2 RNA or protein for long periods of time remain contagious. Very preliminary data suggest that such patients do not infect others. For example, Vibholm et al. (2021) found that ∼5% of their subjects tested positive for SARS-CoV-2 ∼90 days after infection by RT-PCR nasopharyngeal testing, but transmission to close contacts was not observed. However, some Ebola outbreaks appear to have been started by post-Ebola patients harboring persistent reservoirs of the virus (Lo et al., 2017; Virological, 2021). The overall topic of RNA virus persistence and infectivity must therefore be further studied not just for a better understanding of PASC, but for the sake of best managing the COVID-19 pandemic itself.

## Immune Dysregulation Promoted by SARS-CoV-2 Can Lead to Reactivation of Already Acquired Neurotrophic Pathogens Such as Herpesviruses

Another possible scenario for persistent symptom development in some PASC patients is that SARS-CoV-2 may fully clear from patient blood, tissue and nerves after acute infection. However, the virus may dysregulate the host immune response during acute COVID-19 in a manner that allows previously harbored pathogens to reactivate, infect new body sites, and drive new chronic symptoms.

It is well understood that humans accumulate persistent viruses over the course of a lifetime. These viruses generally persist in dormant, latent, or non-cytolytic forms, but may reactivate under conditions of stress or immunosuppression. Indeed, people regarded as healthy have been shown to harbor a wide range of persistent viruses in blood, saliva (Wylie et al., 2014), or tissue that are capable of activation under such conditions (Virgin et al., 2009).

For example, Kumata et al. (2020) took RNA-seq data from the Genomic-Tissue Expression Project: a public resource created to study tissue-specific gene expression/regulation from 51 tissue types collected from 547 healthy individuals at autopsy. They successfully identified 39 viral species in at least one tissue (tissue types included brain, pituitary, esophagus, thyroid, heart, breast, lung, kidney, adrenal gland, prostate, nerve, adipose tissue, blood vessel, ovary, and uterus). Viruses identified in the various tissue samples included Epstein-Barr virus (EBV), herpes simplex virus (HSV-1), varicella zoster virus (VZV), cytomegalovirus (CMV), human herpes virus 6-A/B (HHV6-A/B), human herpes virus 7 (HHV-7), hepatitis C virus (HCV), human papilloma virus (HPV), adeno-associated virus and RNA viruses including respiratory syncytial virus (RSV), and parainfluenza virus 3. Human coronavirus 229-E was identified in brain, thyroid, heart, lung, stomach, adrenal gland, skin and blood samples. The team stated: “We found that the human virome includes several viruses ‘hidden’ by expression/replication in tissues inside the human body without being abundant in the blood.”

Kumata and team also characterized how viruses they identified associated with human gene expression and immune activity. As a general trend, gene expression and immune changes correlated with viral presence in a tissue were associated with components of the immune response known to control pathogen activity. For example, genes associated with “type 1 interferon signaling pathway,” “defense response to virus,” and “viral process” were highly upregulated in hepatitis C virus liver tissue samples.

This suggests that persistent viruses are normally kept “in check” by the host immune system. However, if the immune response is weakened, challenged, or dysregulated, the same viruses may change their gene expression or protein production to drive a range of persistent symptoms. For example, more than 90% of humans harbor at least one strain of herpesvirus (Gacek, 2002), but most infections are kept in latency by host interferons (Decman et al., 2005; Le-Trilling and Trilling, 2015). However, by disabling the host interferon response, (Acharya et al., 2020), SARS-CoV-2 may allow persistent herpesviruses to take advantage of acute COVID-19. Early studies and case histories demonstrate that herpesviruses are indeed reactivating in COVID-19 patients (Chen et al., 2020; García-Martínez et al., 2020). For example, Xu R. et al. (2020) reported VZV and HSV-1 reactivation in a patient with severe COVID, which correlated with the onset of septic shock. Another team demonstrated reactivation of HHV-6, HHV-7, and EBV in patients with acute COVID-19 (Drago et al., 2021).

Herpesvirus infection has been tied to the development of many different chronic disease states. For example, EBV, CMV, and Kaposi’s sarcoma-associated herpesvirus (KSHV) are recognized as cancer-causing or oncogenic viruses (Luo and Ou, 2015). These and related viruses such as hepatitis B virus (HBV), HCV, and papillomavirus can drive diseases like cancer by expressing proteins that directly modulate human gene expression, the human immune response, host cell metabolism, and even the host epigenetic environment to promote a range of pathological processes (Proal et al., 2017).

For example, EBV can express protein EBNA2. One study found that EBNA2 and its related transcription factors can bind and activate human genes associated with the development of dozens of chronic conditions including multiple sclerosis, rheumatoid arthritis, type 1 diabetes, and celiac disease (Harley et al., 2018). In fact, the team demonstrated that EBNA2 directly binds half of the locations on the human genome known to contribute to lupus risk. EBV protein EBNA3 has been shown to bind to the human vitamin D nuclear receptor (VDR) to block activation of its target genes (Yenamandra et al., 2010). Since the VDR controls the expression of hundreds of human genes, including several that regulate key components of the innate immune response such as TLR-2 and the cathelicidin antimicrobial peptides (Wang et al., 2005), this disruption can have far reaching negative consequence for overall host immune function.

Epstein-Barr virus can also hijack the metabolism of the cells it infects. For example, Wang et al. (2019) found that, in infected primary human B cells, EBV upregulated host mitochondrial 1C metabolism. Expression of EBV proteins, and not the host cell innate immune response, was required for this 1C induction. Indeed, all viruses, and many bacterial and fungal pathogens, hijack the metabolism of the cells they infect in order to gain amino acids, lipids, and other substrates required for their own replication and survival (Escoll and Buchrieser, 2018; Thaker et al., 2019; Proal and VanElzakker, 2021). Dozens of human pathogens capable of persistence modulate the activity mitochondrial electron chain complexes (Escoll et al., 2019). This leads to bioenergetic and metabolic alterations in infected host cells that dysregulate oxidative phosphorylation levels and even regulation of cell death.

Persistent viruses that activate under conditions of SARS-CoV-2-driven immunosuppression or immune dysregulation might also infect new body sites and cell types, allowing them to drive new symptoms. Both herpesviruses and enteroviruses are neurotrophic pathogens, with the herpesvirus active life cycle relying on moving through nerves (Steiner et al., 2007; Huang and Shih, 2015). It follows that under conditions of immunosuppression, they can move out of blood, saliva, or tissue and deeper into the CNS. Once in the CNS, such viruses have been shown capable of driving a range of neuroinflammatory processes. For example, HHV6 and HHV7 were recently identified in autopsied Alzheimer’s brains, where they regulated host molecular, clinical, and neuropathological networks in a manner that contributed to inflammation and neuronal loss (Readhead et al., 2018). HHV-6 was shown to accelerate neuroinflammation in a non-human primate model of multiple sclerosis (Leibovitch et al., 2018).

In some cases, even latent viruses express proteins capable of driving chronic symptoms. For example, elevated cytokine expression in response to HSV-infected peripheral nerve ganglia was shown to persist even when the virus remains in a latent, non-replicating state (Chen et al., 2000). Similarly, SITH-1, a protein expressed during HHV-6B latency, has been connected to HPA axis dysregulation and increased risk of depression (Kobayashi et al., 2020).

## SARS-CoV-2 May Impact the Activity of Bacteria, Fungi, and Parasites

Like viruses, many bacterial, fungal, and parasitic pathogens also change their activity and/or infect new tissue and the CNS under conditions of immune dysregulation or stress. These include tick-borne bacterial pathogens such as *Borrelia burgdorferi*, *Rickettsia*, and *Bartonella henselae* (Rupprecht et al., 2008; Sahni and Rydkina, 2009). *Bartonella henselae* can drive blood vessel dysfunction by infecting vascular endothelial cells, so increased activity of the pathogen in patients additionally infected with SARS-CoV-2 could aggravate or sustain long-term vasculature or circulatory symptoms (Beerlage et al., 2012; Balakrishnan et al., 2016).

**FIGURE 3:**
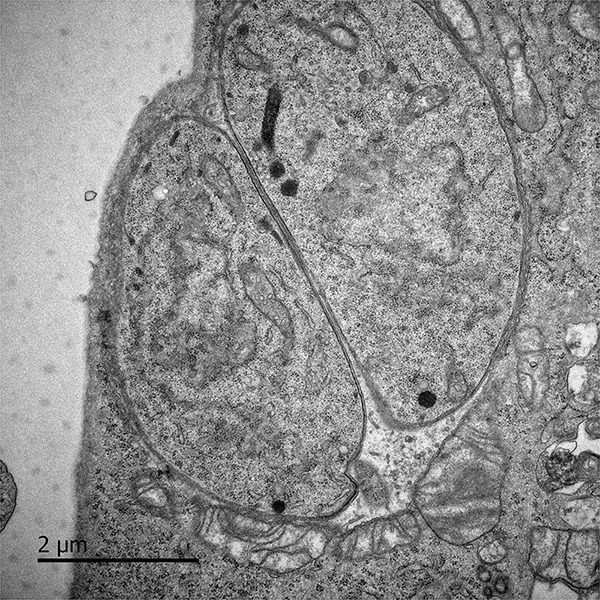
Murine embryonic fibroblasts, 6 h after infection with *Toxoplasma gondii* tachyzoites. Persistent pathogens such as *Toxoplasma* may reactivate during acute COVID-19. Original image courtesy of Dr. Lena Pernas.

Approximately one third of the world’s population harbors *Toxoplasma gondii* (*T. gondii*), a parasite that can differentiate into a latent form that establishes persistent infection in muscle and brain tissue (Montoya and Liesenfeld, 2004; Waldman et al., 2020) (Figure 3). According to CDC estimates, 11% of the United States population 6 years and older have been infected with the persistent parasite (CDC, 2019). A number of research teams have now connected *T. gondii* to the development of conditions such as cancers, epilepsy, Alzheimer’s disease, and schizophrenia (Ngô et al., 2017). Immunosuppression greatly facilitates *T. gondii’s* ability to drive chronic symptoms. For example, *Toxoplasma* reactivation has been reported in patients administered immunosuppressive biologics such as anti-TNF drugs (Baddley et al., 2014; Lewis et al., 2015).

## Functional Redundancy in Pathogen-Driven Processes Can Facilitate Chronic Symptom Development

It is important to understand that different persistent pathogens capable of reactivating and/or infecting new tissue under conditions of SARS-CoV-2 immune dysregulation often modify human gene expression, immunity, and metabolism via similar mechanisms of action. Some even drive disease in a similar fashion to SARS-CoV-2. This functional redundancy means that the activity of one pathogen can support the virulence of the next (a “multiple hit model”) (Proal and Marshall, 2018). For example, SARS-CoV-2 expresses proteins that dysregulate the host interferon response (Acharya et al., 2020). However, viruses such as HCV and HSV also downregulate host interferon signaling to better drive disease (Weber, 2007). Indeed, HSV protein ICP0 directly disrupts interferon signaling by both blocking the JAK–STAT pathway and downregulating expression of interferon-stimulated genes (Katze et al., 2002). It follows that patients already harboring HSV at the time of SARS-CoV-2 infection may have more trouble mounting an immune response that fully clears SARS-CoV-2 from all body sites. Conversely, SARS-CoV-2 may fully clear from a patient during acute disease, but not before promoting an atmosphere conducive to increased long-term interferon dysregulation by HSV.

Different persistent pathogens may also work in concert with SARS-CoV-2 to sustain a hypoxic environment conducive to long-term oxygen, vasculature and/or related metabolic problems in patients with PASC. Hypoxia-inducible factor (HIF-1) is a central regulator of host cell adaptation and response to low oxygen levels (Palazon et al., 2014). In a brain organoid model, elevated HIF-1α staining indicated that SARS-CoV-2 induced a locally hypoxic environment in neural tissues (Song et al., 2020). Many other common viral, bacterial and protozoan pathogens also either directly or indirectly enhance HIF-1α stability in a manner that can sustain a range of chronic disease processes (Werth et al., 2010; Zhu et al., 2016). For example, HIF-1 activation occurs during *Bartonella henselae* infection, and is associated with increased oxygen consumption, cellular hypoxia, and decreased ATP levels in infected host cells (Kempf et al., 2005). A hypoxic environment can, in turn, promote the activity of yet other persistent pathogens in a feed-forward fashion. For example, hypoxia can induce EBV reactivation when HIF-1α binds to EBV’s primary latent-lytic switch gene BZLF1 (Kraus et al., 2017).

The high level of functional redundancy by which different persistent pathogens modulate human gene expression, immunity, and metabolism means that no two patients with chronic symptoms resulting from their activity need harbor the exact same mix of pathogens to develop similar sets of symptoms. The same is true for location of infection. The ability of different persistent pathogens to infect the same cell type, the same tissue, the same brain region, or the same nerve could lead to common symptoms in patients harboring different organisms. For example, we have previously hypothesized that in some patients diagnosed with ME/CFS, viral or bacterial pathogens may infect the vagus nerve, which could lead to similar sets of chronic symptoms in different patients (VanElzakker, 2013).

## The Activity of Persistent Pathogens Can Serve as a Form of Predisposition to COVID-19

It is also worth noting that the activity of persistent pathogens already harbored by a patient infected by SARS-CoV-2 can serve as a form of predisposition for COVID-19 development. That is because the life-long need to control the virulence of such pathogens places a significant burden on the human immune system. For example, Brodin et al. (2015) measured hundreds of immune parameters including cytokine responses, cell population frequencies, and serum proteins in monozygotic twins discordant for CMV infection (each twin pair included one viral seropositive and one viral seronegative sibling). CMV discordant monozygotic twins showed greatly reduced correlations for many immune cell parameters, including effector CD8+ cell and gamma-delta T-cell frequency, cell signaling responses to IL-6 and IL-10 stimulation, and serum concentrations of IL-6 and IL-10. Overall, non-heritable factors determined more than half the variance in 77% of immune parameters dispersed throughout the immune network, and determined more than 80% of the variance within 58% of measured immune parameters. The findings illustrated “how at least one type of microbial exposure can dramatically modulate the overall immune profile of healthy individuals.”

In a separate study, Sylwester et al. (2005) found that approximately 10% of CD4+ and CD8+ memory T cells in CMV seropositive subjects can be directed against the virus. It follows that an existing infection can impact an individual’s immune responses to SARS-CoV-2, and that variability in such immune modulation could additionally influence long-term PASC symptom development.

## Reactivated Viruses and COVID-19 Associated Myocarditis

SARS-CoV-2 appears capable of directly infecting the heart (Bose and McCarthy, 2020). For example, in a human engineered heart tissue model Bailey et al. (2020) showed that SARS-CoV-2 selectively infects cardiomyocytes in a manner that can interfere with heart muscle contraction. Ongoing myocardial inflammation has been reported after recovery from acute COVID-19, even in mildly symptomatic or asymptomatic patients. For example, Rajpal et al. (2021) used cardiac magnetic resonance imaging to demonstrate that 15% of Ohio State University athletes had myocarditis after mild COVID-19. In a separate cohort study of 100 recently recovered COVID-19 patients, cardiac magnetic resonance imaging showed cardiac involvement in 78% of subjects, and ongoing myocardial inflammation in 60% of subjects (Puntmann et al., 2020). The findings were independent of the severity and overall course of acute COVID-19 illness, preexisting conditions, and time from initial diagnosis.

Myocarditis identified after acute COVID-19 may be driven by SARS-CoV-2 or sterile injury to the heart. However, it is worth noting that persistent vasculotrophic virus parvovirus B19, enteroviruses such as coxsackie A/B, and herpesviruses such as HHV6, EBV, and CMV can drive myocarditis (Rose, 2016; Tschöpe et al., 2021). It follows that reactivation of such pathogens may, either collectively or alone, contribute to myocarditis or related cardiac inflammatory issues in some patients with a PASC diagnosis.

## SARS-CoV-2 or Reactivated Pathogens May Induce Pathological Immune Cell Signaling or Prime Glia

In cases where persistent reservoirs of SARS-CoV-2 or the activity of other pathogens might contribute to some PASC symptoms, such pathogens would be expected to persist as a “low biomass” infection, in which a relatively small number of host cells are infected. This is especially likely of ongoing CNS infection.

However, a low biomass infection can drive serious inflammatory symptoms by activating host immune and metabolic signaling cascades in a feed-forward fashion. Mast cells and glial cells are particularly well-studied for their ability to amplify immune signaling cascades associated with low-biomass infection or inflammatory insult. Both cell types play vital roles in the innate immune response toward infection or injury. Upon activation by pathogens, toxins, allergens, or injury, mast cells degranulate and release multiple proinflammatory substances and lipid mediators that can promote inflammatory symptoms. For example, mast cells respond directly to influenza A virus infection by releasing proteases, histamine, leukotrienes, antiviral chemokines, inflammatory cytokines and other mediators in an effort to control the virus (Graham et al., 2015). However, if viral load becomes too high, or the infection cannot be fully contained, the same mast cells can drive a pathological immune response.

In the central nervous system, mast cells act in close concert with microglia – the brain’s resident macrophage-derived innate immune cells (Silver and Curley, 2013). When microglia or other glial cells detect infection, injury, or inflammatory mediators, they enter a state of activation in which they change morphology and release their own neuroexcitatory inflammatory mediators. After activating, they retain a “primed” functional state which causes an even more robust response to subsequent challenges.

Glia and mast cell inflammatory signaling cascades are consequently highly sustained by exposure to “multiple hits” (different inflammatory events that collectively amplify their signaling). For example, mast cells activate in response to SARS-CoV-2, but also play a central role in host defense against herpes simplex virus infection via production of TNF-α and IL-6 (Aoki et al., 2013). *Borrelia burgdorferi* spirochetes additionally induce mast cell activation and cytokine release (Talkington and Nickell, 1999). Most forms of sterile tissue injury also result in increased mast cell activity. Thus, any PASC patient with multiple ongoing inflammatory issues would be expected to suffer from increased mast cell and glia-related immunopathology. This “primed” state may also be an important part of symptoms like sensory sensitivity in some individuals who have survived an acute neuroinflammatory event such as encephalitis or concussion, or who may have low levels of persisting or latent neurotropic pathogens.

## SARS-CoV-2 May Dysregulate Host Microbiome/Virome Balance by Facilitating Pathobiont Virulence

Immune dysregulation driven by SARS-CoV-2 might also promote the collective imbalance of the human body’s microbial and viral ecosystems in a manner that could result in PASC symptoms. Humans harbor vast communities of bacteria, viruses, fungi, and archea in many body sites including the gut, urinary tract, pancreas, lungs, and oral cavity (Moffatt and Cookson, 2017; Neugent et al., 2020; Thomas and Jobin, 2020). The bacterial, fungal, and archeal components of these ecosystems comprise the human microbiome, with viral communities collectively referred to as the human virome (Scarpellini et al., 2015).

Even human blood has been shown to harbor communities of organisms, especially in immunocompromised individuals (Olde Loohuis et al., 2018). For example, Kowarsky et al. (2017) used massive shotgun sequencing of circulating cell-free DNA to identify over 3,000 bacteria, viruses, and fungi in blood samples obtained from immunocompromised human patients. The team was forced to add new branches to the “tree of life” to classify many of the organisms. They concluded that the newly discovered microbes “may prove to be the cause of acute or chronic diseases that, to date, have unknown etiology.”

Under conditions of health, these host microbiome/virome communities are kept “in check” by a robust host immune response, and persist in a state of balance or homeostasis. However, dozens of chronic conditions are now tied to dysbiosis: a collective imbalance of microbiome/virome ecosystem composition and dynamics (Belizário and Faintuch, 2018). Conditions characterized by dysbiosis in various body sites include gastrointestinal disorders such as irritable bowel syndrome, Crohn’s disease, and ulcerative colitis, but also a wide range of neuroinflammatory and metabolic disorders such as ME/CFS, Parkinson’s disease, and type 1 and 2 diabetes (Carding et al., 2015; Proal and Marshall, 2018; Baldini et al., 2020). Microbiome/virome dysbiosis of the gut and oral cavity is even being connected to the development of neurological disease states including anxiety, depression, autism spectrum disorder, and “brain fog”-type symptoms (Rogers et al., 2016; Yang et al., 2019; Cryan et al., 2020).

Microbiome/virome dysbiosis is often characterized by significant shifts in organism community composition and diversity that may favor the growth of opportunistic pathogens. For example, alpha-diversity of the alveolar lung microbiome is significantly decreased in patients positive for *Mycobacterium tuberculosis* (Hu et al., 2020). However, dysbiosis also occurs when existing commensal members of host ecosystems change their gene expression in a manner that increases their virulence. These shifts in virulence can occur on a large-scale, because nearly all bacterial, viral, and fungal organisms in human microbiome/virome communities are pathobionts: they are capable of changing their gene expression to act as pathogens under conditions of imbalance and immunosuppression (Hornef, 2015).

For example, ∼30% of humans harbors *S. aureus* as a member of the normal nasal microbiome (Krismer et al., 2017). However, under conditions of immunosuppression, *S. aureus* can change its gene expression to drive a range of diseases, from skin infections to life-threatening conditions such as meningitis and endocarditis (Lucas et al., 2013). Another study demonstrated that “commensal” *E. coli* could evolve into virulent clones that escape phagocytosis in less than 500 generations (Miskinyte et al., 2013). For many microbes, this evolution toward pathogenicity occurs via gain of function mutations or alteration of the current genome in a manner that results in gene loss (Hottes et al., 2013). For example, loss of the gene *mucA* increases *Pseudomonas aeruginosa’s* ability to resist pulmonary clearance and evade phagocytosis.

By dysregulating or disabling the immune response, newly infecting pathogens such as SARS-CoV-2 may promote this increased pathobiont virulence and general microbiome/virome dysbiosis. For example, the influenza virus weakens the host immune response via various mechanisms, including impairment of macrophage and neutrophil function, depletion of alveolar macrophages, and disruption of respiratory tract mucosal barrier function (Ghoneim et al., 2013). A study in mice found that infection with H1N1 influenza virus drove a shift in the composition and diversity of the lower respiratory tract (LRT) microbiome (Gu et al., 2019). Specifically, influenza virus infection modulated the dominant LRT microbiome bacterial class, which was followed by an increase in the relative abundance of respiratory pathobionts *Streptococcus* and *Staphylococcus*. This LRT dysbiosis did not normalize even in the recovery phase of the infection.

Several studies suggest that COVID-19 may promote microbiome/virome dysbiosis in a similar fashion. Shen et al. (2020) found that the bronchoalveolar lavage fluid microbiome of COVID-19 patients showed enrichment of pathogenic bacteria and elevated levels of oral and upper respiratory commensal bacteria. In another pilot study, the gut microbiome of hospitalized COVID-19 patients was characterized by enrichment of opportunistic pathogens such as *Actinomyces viscosus*, and depletion of commensal species (Zuo et al., 2020). This dysbiosis persisted even after resolution of respiratory symptoms and SARS-CoV-2 clearance.

However, the above COVID-19 studies must be interpreted to account for the fact a dysbiotic microbiome/virome community can also impact initial SARS-CoV-2 risk and virulence. That is because the composition and activity of microbiome/virome communities in any body site can influence initial host susceptibility and ongoing control of infecting pathogens like SARS-CoV-2. Organisms in microbiome/virome communities contribute to host defense by priming the immune system to better manage pathogen attack, producing compounds that disable pathogens, or by simply occupying ecosystem niche space in a manner that prevents pathogen colonization of a tissue or body site (Eastment and McClelland, 2018; Cheng et al., 2019). Dysbiosis of a microbiome/virome community can disrupt these protective functions.

In the lung microbiome, organisms inhabiting the respiratory surface can act as a barrier, preventing the attachment of invading pathogens to host cells (Khatiwada and Subedi, 2020). A loss of diversity, which is often associated with dysbiosis, can deplete this protective barrier. Some research also indicates that ongoing surveillance of the commensal lung microbiome by macrophage, T cells, dendritic cells, and other immune cells may prime the lung immune system in a manner that helps keep lung pathogens and pathobionts “in check” (Ramírez-Labrada et al., 2020).

Composition and activity of the oral and nasal microbiome/virome may have a particularly strong impact on COVID-19 sequelae since a high proportion of organisms in the respiratory tract microbiome are derived from oral and nasal organisms that can “shed” into the lungs (Xiang et al., 2021). Underlying conditions tied to oral microbiome dysbiosis also appear to increase COVID-19 severity. For example, *Porphyromonas gingivalis* (*P. gingivalis*) and related oral pathogens can promote collective dysbiosis of the oral microbiome in a manner that leads to gum disease periodontitis (Yost et al., 2015). In a study of 568 COVID-19 patients, Marouf et al. (2021) found that subjects with periodontitis were 3.5 times more likely to be admitted to intensive care, 4.5 times more likely to require assisted ventilation, and had an almost 9 times higher risk of death then subjects without periodontitis (Marouf et al., 2021). Levels of white blood cells, C reactive protein, and D-dimer (indicative of blood clotting) were also significantly higher in the blood of COVID-19 subjects with periodontitis.

## Microbiome Dysbiosis Can Disrupt Host Metabolic and Neuroendocrine Signaling and/or Epithelial Barrier Function

Microbiome/virome dysbiosis can also have a profound impact on host immune, metabolic and hormonal signaling. Humans harbor trillions of organisms in their microbiome/virome ecosystems (Ursell et al., 2012). Indeed, the human body harbors more microbial cells than human cells (Sender et al., 2016). Since all such organisms produce proteins and metabolites as part of their active lifecycles, the majority of proteins and metabolites in the human body are not “self,” but produced or modified by the microbiome/virome. For example, Wikoff et al. (2009) documented a large effect of the gut microbiome on murine blood metabolites including antioxidants, amino acids, and toxins.

Human bacteria have been shown to produce and/or consume a wide range of mammalian neurotransmitters, including norepinephrine, dopamine, serotonin, and gamma-aminobutyric acid (GABA) (Galland, 2014; Strandwitz, 2018). Human immune cell activity is also modulated by the microbiome/virome-derived proteins and metabolites. For example, in an experimental mouse model, Rothhammer et al. (2018) found that tryptophan created by gut microbiome bacteria interacted with the AHR receptor on microglia/astrocytes. Subsequent transcriptional changes regulated communication between the two cell types. A separate study found that metabolites produced by *Clostridium orbiscindens* protect mice from influenza via augmentation of interferon-I signaling (Steed et al., 2017).

It follows that microbiome/virome dysbiosis can disrupt the homeostasis of host signaling pathways in a manner that might impact chronic disease development. For example, Tetz et al. (2018) identified changes in the Parkinson’s disease gut bacteriophage community. These included shifts in the bacteriophage/bacteria ratio of bacteria known to produce dopamine – the neurotransmitter involved in Parkinson’s pathology (Masato et al., 2019).

Microbiome/virome dysbiosis is also often accompanied by inflammation that can lead to dysfunction or breakdown of oral barriers such as the gingiva, the epithelial lining of the gut, or related barriers (Shin and Kim, 2018). This increased epithelial or oral barrier permeability allows pathogens/pathobionts in such communities to translocate into the blood, where their presence and products can sustain a range of systemic inflammatory processes. For example, Adams et al. (2019) found that Parkinson’s blood contained increased bacterial LPS, associated cytokines, and gingipain proteins derived from oral pathogen *P. gingivalis*. These inflammagens increased blood hypercoagulation, amyloid formation, and induced profound ultrastructural changes to platelets.

**FIGURE 4:**
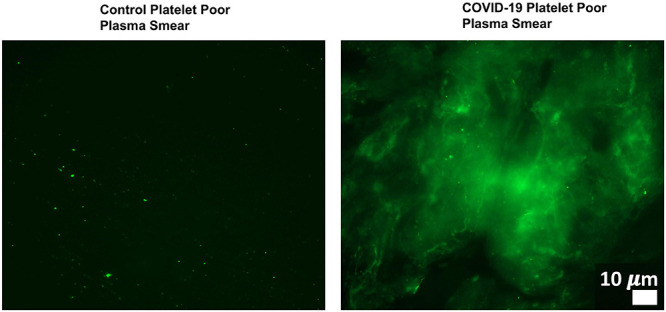
Microclots are significantly increased in COVID-19 plasma as compared to controls. Fluorescence microscopy of COVID-19 and control platelet poor plasma incubated with the dye thioflavin (ThT), which detects spontaneously formed microclots. Original image courtesy of Dr. Resia Pretorius.

Changes in host signaling or epithelial barrier permeability due to microbiome/virome dysbiosis could contribute to PASC symptoms. Redundancy in the molecular mechanisms involved in such processes and SARS-CoV-2’s own activity would result in more severe pathology. For example, Adams et al. (2019) showed that *P. gingivalis* and bacteria translocated into blood can drive hypercoagulation. However, SARS-CoV-2 also secretes products/proteins that modulate blood clotting cascades (Eslamifar et al., 2020). Pretorius et al. (2020a, b, 2021) identified fibrinolytic resistant amyloid microclots in COVID-19 plasma (Figure 4) and in plasma samples collected from 11 PASC subjects. This suggests that patients who suffer from both issues/insults might be disproportionally impacted by PASC symptoms that could result from blood inflammatory/coagulation cascades.

## SARS-CoV-2 May Promote Extended “Autoantibody” Production, Often via Molecular Mimicry

Another mechanism by which SARS-CoV-2 may promote PASC symptoms is by activating the host immune response in a manner that leads to long-term autoantibody production. Several research teams have isolated a range of autoantibodies in acute COVID-19 patients. Wang et al. (2020) used a high-throughput autoantibody discovery technique called Rapid Extracellular Antigen Profiling (REAP) to screen a cohort of 194 SARS-CoV-2 infected COVID-19 patients for autoantibodies against 2,770 extracellular and secreted proteins (the exoproteome). COVID-19 subjects exhibited dramatic increases in a wide range of autoantibody reactivities compared to uninfected controls.

Identified autoantibodies targeted proteins involved in a wide range of immunological functions including interferon responses, type II immunity, acute phase response, leukocyte trafficking, and lymphocyte function/activation. A high prevalence of tissue-associated autoantibodies were also observed in COVID-19 subjects. These included autoantibodies directed against vascular cell types (e.g., endothelial adhesion molecule PLVAP, regulator of angiogenesis RSPO3), against the CNS compartment (e.g., metabotropic glutamate receptor, orexin receptor HCRT2R) and against connective tissue and extracellular matrix targets (e.g., matrix metalloproteinases MMP7 and MMP9, suspected regulator of cartilage maintenance OTOR), among others. Many autoantibodies identified in the study displayed functional activity and could be correlated with various immunological, virological, and clinical parameters within COVID-19 patient samples.

Autoantibody production in the study’s COVID-19 subjects could have resulted from aberrant immune responses under conditions of pro-inflammatory disease. However, the data must also be interpreted via the activity of organisms/pathogens/pathobionts harbored by subjects at the time of SARS-CoV-2 infection. That is because “autoantibodies” in human disease often arise as a result of molecular mimicry, or sequence homology, between host and organism peptides and proteins (Cusick et al., 2012). Indeed, the structures of a vast range of viral, bacterial and fungal proteins/metabolites are identical or very similar to those expressed by their human hosts. For example, Kusalik et al. (2007) found that pentamers from the hepatitis C virus polyprotein have a high level of structural similarity to 19,605 human proteins (57.6% of the human proteome).

Human antibodies are also notoriously polyspecific – they have flexible specificity and are able to recognize multiple antigens with similar conformations (Wing, 1995). Even the T cells that help direct antibody responses are characterized by flexible specificity (Davis, 2015). It follows that when the human immune system creates an antibody to target a pathogen/organism protein, the same antibody may additionally target a human protein with similar structural characteristics. This cross-reactivity can result in collateral damage, inflammation, and the production of what are often deemed “autoantibodies.” If multiple organisms become involved in this immune system “cross-fire” due to molecular mimicry, multiple “autoantibodies” may be generated.

SARS-CoV-2 itself has been shown to drive cross-reactive antibody responses. For example, Kreye et al. (2020) identified high-affinity SARS-CoV-2-neutralizing antibodies that cross-reacted with gut, kidney, lung, heart, and brain mammalian self-antigens. Antibody binding in the brain occurred in the basal ganglia, hippocampal formation, olfactory bulb, and cerebral cortex (Kreye et al., 2020). Another study found that SARS-CoV-2 proteins can share homology with neuron protein epitopes found within vagus or brainstem nuclei such as the jugular ganglion, nodose ganglion, dorsal motor nucleus, and nucleus ambiguus (Marino Gammazza et al., 2021). However, under conditions of inflammation, other organisms/pathogen/pathobionts harbored by COVID-19 patients may also contribute to “autoantibody” production.

For example, Doxey and McConkey (2013) developed and used a computational pipeline to analyze the proteomes of 66 non-pathogenic and 62 pathogenic bacterial species. They screened for top pathogen-specific or pathogen-enriched sequence similarities to human proteins and identified hundreds of potential molecular mimicry relationships. Examination of statistically enriched functions, homology to virulence factors, and comparison with the literature further demonstrated that the identified bacterial mimics target key host structures. These included collagen and the extracellular matrix, and multiple signaling pathways such as cell adhesion, lipid metabolism, and immune signaling.

Another study tested subjects for the presence of IgA or IgG autoantibodies directed against 14 important regulatory peptides including vasopressin, insulin, ghrelin, and leptin. Many cases of sequence homology were identified between these peptides and proteins created by bacterial and fungal organisms common in gut microbiome ecosystems such as *Lactobacilli*, *Escherichia coli, Helicobacter pylori*, and *Candida* species (Fetissov et al., 2008). Immunoglobulins secreted by B cells infected with EBV have been shown capable of reacting with dozens of human antigens including albumin, thyroglobulin, renin (Seigneurin et al., 1988). EBV can also express protein EBNA-1, antibodies against which cross-react with lupus-associated autoantigens such as Sm D1, Ro, and Sm B/B′ (Poole et al., 2006).

With such findings in mind, at least some “autoantibody” production in COVID-19 patients may result from the following sequence of events: When a patient is exposed to SARS-CoV-2, activated innate and adaptive immune cells move deeper into infected tissue. As the immune cells surveil the tissue, B cells produce antibodies against not just SARS-CoV-2, but also against other organisms/pathogens/pathobionts in the same niche. If such organisms create proteins and metabolites with homology to human proteins, “autoantibodies” may be generated in response to their presence. This is especially likely to happen if SARS-CoV-2 infects a body site with a dense microbiome/virome ecosystem and high pathobiont activity.

Under such conditions, “autoantibody” production would vary widely between different COVID-19 patients. That is because the composition and virulence of patient microbiome/virome communities capable of contributing to cross-reactive “autoantibody” production differs greatly among individuals. The same is true of persistent pathogens capable of reactivation in COVID-19 tissue. Moreover, SARS-CoV-2 infects different body sites and cell types in different patients.

This model fits with the Wang et al. (2021) COVID-19 “autoantibody” findings. The team was unable to identify COVID-19 “autoantibody” responses that could extensively partition patients into specific phenotypes or outcomes. Instead, they observed an extensive constellation of rare and uncommon “autoantibody” reactivities with large apparent effect sizes. This led to the conclusion that “relatively private reactivities are common in COVID-19, and the aggregate sum of these multifarious responses may explain a significant portion of the clinical variation in patients.” In some patients, this varied “autoantibody” production might continue after resolution of acute COVID-19 disease, leading to PASC symptoms.

## SARS-CoV-2 and/or Related Inflammatory Insults May Disrupt Brainstem Signaling

**FIGURE 5:**
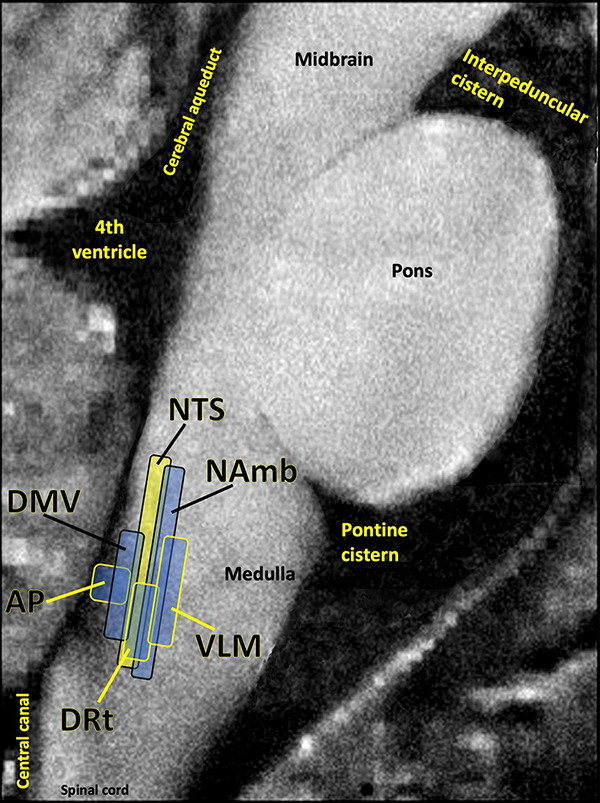
Several studies have shown that the dorsal brainstem can be affected or infected by SARS-CoV-2. This region is dense with nuclei that may be important for PASC. AP, area postrema, a circumventricular organ lacking a blood brain barrier, involved in nausea/vomiting; DRt, dorsal reticular nucleus, involved in inflammatory pain; DMV, dorsal motor nucleus of the vagus nerve, site of parasympathetic neurons and the main source of the vagal innervation of trunk organs; NAmb, nucleus ambiguus, contains soma of cholinergic preganglionic parasympathetic neurons that control heart rate; NTS, nucleus of the solitary tract, site of sensory vagus nerve soma that respond to detection of peripheral inflammation; VLM, ventrolateral medulla, involved in noradrenergic sympathetic nervous system signaling, autonomic control of blood pressure and breathing. Image adapted with permission from Sclocco et al. (2018). Original image courtesy of Dr. Zahra Mona Nasiriavanaki, Paulita Lara Mejia, and Dr. Roberta Sclocco.

Many patients given a PASC diagnosis report a spectrum of symptoms that either meet the diagnostic criteria for ME/CFS, or are very similar in nature to those suffered by ME/CFS patients. These symptoms include dysautonomia, diffuse pain, sleep problems, flu-like symptoms, trouble concentrating, and nausea. The central role of the brainstem in the sickness behavior response, autonomic control, and arousal suggests that dysfunctional brainstem signaling may be an important driver of PASC symptoms that overlap with those of ME/CFS. Indeed, the dorsal brainstem is packed with nuclei governing symptoms such as dysautonomia, sleep problems, nausea, pain, and sickness (Figure 5). Several studies have reported functional and structural brainstem abnormalities in ME/CFS (Barnden et al., 2019; VanElzakker et al., 2019; Shan et al., 2020), including one study demonstrating brainstem glial activation positively correlated with cognitive impairment (Nakatomi et al., 2014).

One of the best-replicated neurological findings upon COVID-19 autopsy is immune activation in the brainstem, including activated astrocytes and microglia, and infiltration of cytotoxic T cells (Solomon, 2021). As previously described, autopsy studies also indicate that SARS-CoV-2 may directly invade the brainstem in acute COVID-19 (Matschke et al., 2020; Meinhardt et al., 2021). In some COVID-19 patients either this brainstem inflammation or infection may persist, driving PASC symptoms.

However, brainstem signaling is also strongly impacted by infections and inflammatory events that occur outside the brain itself. One meta-analysis of 457 participants in 24 peripheral immune challenge neuroimaging studies found the brainstem to be a central hub in the inflammation neurocircuitry (Kraynak et al., 2018). This is because discrete brainstem nuclei such as the nucleus of the solitary tract (NTS) and the parabrachial nuclei transmit inflammatory signaling from the periphery to the limbic system and neocortex. The NTS in the dorsal brainstem contains a majority of the cell bodies of the sensory (afferent) vagus nerve, the 10th cranial nerve. Vagus is a long, highly branched nerve that densely innervates every major trunk organ including the pancreas, liver, spleen, heart and bladder, along with the gastrointestinal lining and lymph nodes (Kenny and Bordoni, 2019). Vagus has particularly dense innervation of tissues like the lung and esophagus, both of which sample external air and are dense with ACE2 receptors (Xu H. et al., 2020).

The terminals of the sensory vagus nerve contain chemoreceptors that detect proinflammatory cytokines and other paracrine inflammatory mediators that do not normally transverse the blood–brain barrier. Upon detection of peripheral immune mediators, neuroimmune signaling into the NTS triggers a “mirror response” of glial activation in the dorsal brainstem that causes the “sickness behavior response” – a neurological and behavioral component of the innate immune response. This involuntary sickness response includes fatigue, fever, myalgia, and other symptoms that overlap with those of ME/CFS (VanElzakker, 2013) and PASC.

Any insult capable of driving ongoing proinflammatory cytokine production in a body site innervated by the vagus nerve can initiate or perpetuate this sickness behavior response and associated chronic symptoms. These include tissue injury, persistent SARS-CoV-2, the activity of other pathogens, immune cells activated via molecular mimicry signaling, or microbiome/virome dysbiosis. It follows that no two PASC patients suffering from ongoing activation of the vagus nerve-to-NTS neuroimmune signaling pathway need harbor the exact same mix of proinflammatory drivers to develop similar sets of chronic symptoms.

Interestingly, nearly overlapping with the NTS is the area postrema – a circumventricular organ that lacks a blood–brain barrier to large molecules and is relatively dense with mast cells (Porzionato et al., 2004), especially at its border with the NTS. Circumventricular organs are sites where cytokines and peripheral immune cells can directly enter the central nervous system via blood, where they can trigger the same mirror response of glial activation that is triggered by the neuronal signaling of the vagus nerve. Area postrema is a central hub for subjective nausea (Horn et al., 2014), a common symptom in PASC and ME/CFS. Also, the initial sites of the glial cell mirror response such as NTS and area postrema are physically near potentially PASC-relevant nuclei governing pain and autonomic regulation (Figure 5). Additionally, activation of central nervous system glia is a necessary step in neuroinflammation, including further loosening of the blood–brain barrier.

COVID-19 is associated with a profound escalation in clotting cascades (Grobler et al., 2020) including an amyloid form of fibrin that may be associated with infection-related systemic inflammatory conditions (Esposito et al., 2016; Pretorius et al., 2020b). This may affect the meninges, the three membrane layers surrounding the central nervous system. Because the blood vessels of the dura mater layer do not feature a blood–brain barrier, some clotting-associated compounds such as fibrinogen and fibrin can leak into the dura mater (Rohde, 2012), which may affect dural function. Indeed, fibrin shows such affinity for adhesion with the dura mater that fibrin sealants are widely used in neurosurgery to seal dural sutures (Esposito et al., 2016). Furthermore, fibrinogen specifically induces a sustained glial response at the perivascular spaces surrounding neurovasculature, a process worsened by blood–brain barrier disruption during neuroinflammation (Davalos et al., 2012). Enlarged and numerous perivascular spaces lined with activated immune cells are a common feature in COVID-19 autopsies (Kantonen et al., 2020; Lee et al., 2021). Cerebrospinal fluid flow and intracranial pressure regulation rely on the elasticity of meninges and perivascular spaces (Brinker et al., 2014).

Due to its location between the 4th ventricle and foramen magnum, the dorsal brainstem is also uniquely vulnerable to alterations in cerebrospinal fluid pressure. An ongoing area of research centers on whether long-term illness initiated by acute infection may be associated with variance in the craniocervical junction (Rowe et al., 2018; Bragée et al., 2020) or sacral spinal cord structure (Mantia et al., 2015). Such an association could reflect a pre-existing vulnerability factor, or a consequence of pathology related to acute illness effects on cerebrospinal fluid pressure, infection, or inflammatory effects on the elasticity of the meninges or laxity of craniocervical structural ligaments.

Many successful human pathogens that infect tissues upregulate host matrix metalloproteinases (MMPs) (Sellner et al., 2006; Singh et al., 2012). MMPs are enzymes that impact tissue remodeling and neovascularization via degradation of extracellular matrix proteins such as collagen and fibronectin (Gebbia et al., 2001). Ongoing research is investigating whether certain pathogens interact with connective tissue disorder risk genes to accelerate connective tissue damage, and whether damage to the meninges or craniocervical ligament connective tissues could contribute to chronic illness. SARS-CoV-2 itself has been shown to upregulate MMP expression (Ueland et al., 2020). Pablos et al. (2020) found that having a connective tissue or chronic condition such as lupus, scleroderma or vasculitis doubled the severity of COVID-19 infection. Also, it is worth noting that a subset of patients in the Wang et al. (2020) COVID-19 autoantibody study created “autoantibodies” against connective tissue and extracellular matrix targets, including matrix metalloproteinases MMP7, MMP9, and other tissue-associated antigens (Wang et al., 2020).

## Certain Human Genome Variants and/or Herv Activity May Facilitate Pasc Development

The unique human genes harbored by a given PASC patient may predispose to how environmental insults, including infection with SARS-CoV-2 itself, are able to drive disease and potential chronic symptoms. An important research consideration for PASC cases is the identification of specific human genome individual variants whose activity may be particularly detrimental for environment-based sequelae also implicated in PASC development.

For example, genetic variations in innate immune components such as mannose-binding lectin 2 and toll-like receptors play central roles in the immune system’s ability to recognize viruses such as SARS-CoV-2, and initiate an early immune response to fully clear the pathogen (Darbeheshti et al., 2021). If a COVID-19 patient harbors a detrimental mannose binding lectin 2/toll-like receptor genetic variant, they may consequently be at higher risk for PASC symptoms resulting from possible persistence of the virus in tissue.

As information is gained on environment-based factors that contribute to PASC cases, it may be possible to “work backward” from such findings to identify individual human genetic variants that most matter in PASC. For example, if clotting or coagulation issues initiated or driven by SARS-CoV-2 contribute to some PASC cases, then variants in human genes such as leiden factor V that also impact human clotting (Bertina, 1997) could be increasingly measured and studied in patients with a PASC diagnosis.

Acute COVID-19 disease has also been shown to impact the activity of human endogenous retroviruses (HERVs) – DNA sequences of retrovirus origin that the human genome has acquired over the last 100 million years via multiple integrations by now-extinct exogenous retroviruses (Grandi and Tramontano, 2018). HERVs are activated during and participate in inflammatory processes. For example, Kitsou et al. (2020) searched for HERV dysregulation signatures in COVID-19 patients and identified upregulated expression of multiple HERV families in bronchial alveolar lavage samples. HERV-W was the most highly upregulated family among those analyzed by the study. Separately, upregulation of HERV activity is associated with growing number of chronic disease processes (Dolei et al., 2019). For example, HERV-W activity has been connected to MS development (Arru et al., 2007). Indeed, the HERV-W envelope protein has been shown to bind TLR-4 on microglia, triggering the cells to secrete inflammatory cytokines, and preventing such cells from scavenging myelin debris (Madeira et al., 2016).

## Discussion

Across the globe, a subset of patients who sustain an acute SARS-CoV-2 infection are developing a wide range of persistent symptoms that do not resolve over the course of many months (Huang Y. et al., 2021). Patients who develop chronic symptoms after acute COVID-19 are being given the diagnosis Long COVID or post-acute sequelae of COVID-19 (PASC). It is likely that individual patients with a PASC diagnosis have different underlying biological drivers of their symptoms, none of which are mutually exclusive. Potential contributors to PASC include injury to one or multiple organs, persistent reservoirs of SARS-CoV-2 in certain tissues, re-activation of neurotrophic pathogens such as herpesviruses under conditions of COVID-19 immune dysregulation, SARS-CoV-2 interactions with host microbiome/virome communities, clotting/coagulation issues, dysfunctional brainstem/vagus nerve signaling, ongoing activity of primed immune cells, and autoimmunity due to molecular mimicry between pathogen and host proteins.

Overall, it is important to consider that SARS-CoV-2 does not infect a sterile body. The microbiome/virome communities and persistent pathogens a patient harbors at the time of infection (including those inhabiting ecosystems in the gut, mouth, and lungs) may partly impact the viruses’ ability to successfully proliferate. Conversely, immune dysregulation driven by SARS-CoV-2 may disrupt microbiome/virome ecosystem balance or promote the reactivation of already acquired neurotrophic pathogens such as herpesviruses in a manner that can drive a wide range of persistent symptoms. Any ongoing infectious or inflammatory insult that drives afferent vagus nerve neuroimmune signaling can activate a mirror response of glial activation in the dorsal brainstem, with an associated sickness behavior response and changes in autonomic signaling.

Differences in PASC symptom clusters may shed light on biological contributors to individual PASC cases. For example, PASC patients who were asymptomatic during acute COVID-19 disease, and who experience relapsing/remitting chronic symptoms, may be more likely to harbor persistent reservoirs of SARS-CoV-2 in tissue. Alternatively, PASC patients that develop chronic symptoms after hospitalization for acute COVID-19 may be more likely to suffer from injury to one or more body sites.

Because a wide range of biological factors may contribute to PASC development, research on patients with a PASC diagnosis must involve collaboration between teams with a wide range of skillsets including pathology, virology, immunometabolism, neuroscience, and physical therapy/rehabilitation. Research teams that search for SARS-CoV-2 or neurotrophic pathogens that may reactivate under conditions of acute COVID-19 should keep in mind that such pathogens rarely persist in blood, but are instead generally identified in tissue or nerves. Infection of animals or organoid models with the SARS-CoV-2 virus or other neurotrophic pathogens for extended periods of time may provide additional clarity on PASC development. Studies of microbiome/virome activity in PASC would benefit from transcriptome/gene ontology analyses that best capture increased pathobiont virulence. Research on autoimmunity in PASC should include efforts to screen potential identified “autoantibodies” for sequence homology to microbiome/virome-derived proteins/metabolites. Neuroimaging studies that capture brainstem inflammation, compression and indicators of autonomic function in PASC patients are also warranted.

The individualized nature of PASC symptoms also means that different therapeutic approaches may be required to best treat patients with the diagnosis. Identification of individual human genome variants that may augment environment-driven contributions to PASC would bolster such an effort. Overall, an individualized treatment approach to PASC could contribute to a growing era of personalized and predictive/preventative global medicine.

## Author Contributions

AP and MV conceived of and conceptualized the work. AP and MV drafted the article and critically revised the article. Both authors discussed the literature review and contributed to the final manuscript.

## Conflict of Interest

The authors declare that the research was conducted in the absence of any commercial or financial relationships that could be construed as a potential conflict of interest.

## References

[B1] Acharya D. Liu G. Q. Gack M. U. (2020). Dysregulation of type I interferon responses in COVID-19. *Nat. Rev. Immunol.* 20 397–398. 10.1038/s41577-020-0346-x 32457522 PMC7249038

[B2] Adaken C. Scott J. T. Sharma R. Gopal R. Dicks S. Niazi S. (2021). Ebola virus antibody decay–stimulation in a high proportion of survivors. *Nature* 590 468–472. 10.1038/s41586-020-03146-y 33505020 PMC7839293

[B3] Adams B. Nunes J. M. Page M. J. Roberts T. Carr J. Nell T. A. (2019). Parkinson’s disease: a systemic inflammatory disease accompanied by bacterial inflammagens. *Front. Aging Neurosci.* 10:210. 10.3389/fnagi.2019.00210 PMC6718721 31507404

[B4] Aid M. Abbink P. Larocca R. A. Boyd M. Nityanandam R. Nanayakkara O. (2017). Zika virus persistence in the central nervous system and lymph nodes of rhesus monkeys. *Cell* 169 610–620.e14. 28457610 10.1016/j.cell.2017.04.008 PMC5426912

[B5] Aoki R. Kawamura T. Goshima F. Ogawa Y. Nakae S. Nakao A. (2013). Mast cells play a key role in host defense against herpes simplex virus infection through TNF-α and IL-6 production. *J. Invest. Dermatol.* 133 2170–2179. 10.1038/jid.2013.150 23528820

[B6] Arbour N. Côté G. Lachance C. Tardieu M. Cashman N. R. Talbot P. J. (1999). Acute and persistent infection of human neural cell lines by human coronavirus OC43. *J. Virol.* 73 3338–3350. 10.1128/jvi.73.4.3338-3350.1999 10074188 PMC104098

[B7] Arru G. Mameli G. Astone V. Serra C. Huang Y.-M. Link H. (2007). Multiple sclerosis and HERV-W/MSRV: a multicentric study. *Int. J. Biomed. Sci.* 3 292–297. 23675056 PMC3614662

[B8] Baddley J. W. Winthrop K. L. Chen L. Liu L. Grijalva C. G. Delzell E. (2014). Non-viral opportunistic infections in new users of tumour necrosis factor inhibitor therapy: results of the SAfety assessment of biologic ThERapy (SABER) study. *Ann. Rheum. Dis.* 73 1942–1948. 10.1136/annrheumdis-2013-203407 23852763 PMC4273901

[B9] Baggen J. Thibaut H. J. Strating J. R. P. M. Van Kuppeveld F. J. M. (2018). The life cycle of non-polio enteroviruses and how to target it. *Nat. Rev. Microbiol.* 16 368–381. 10.1038/s41579-018-0005-4 29626210

[B10] Bailey A. L. Dmytrenko O. Greenberg L. Bredemeyer A. L. Ma P. Liu J. (2020). SARS-CoV-2 infects human engineered heart tissues and models COVID-19 myocarditis. *bioRxiv* [Preprint] 10.1101/2020.11.04.364315 33681537 PMC7909907

[B11] Balakrishnan N. Ericson M. Maggi R. Breitschwerdt E. B. (2016). Vasculitis, cerebral infarction and persistent Bartonella henselae infection in a child. *Parasit. Vectors* 9:254. 10.1186/s13071-016-1547-9 PMC4862072 27161220

[B12] Baldini F. Hertel J. Sandt E. Thinnes C. C. Neuberger-Castillo L. Pavelka L. (2020). Parkinson’s disease-associated alterations of the gut microbiome predict disease-relevant changes in metabolic functions. *BMC Biol.* 18:62. 10.1186/s12915-020-00775-7 PMC7285525 32517799

[B13] Bannister B. A. (1988). Post-infectious disease syndrome. *Postgrad. Med. J.* 64 559–567. 10.1136/pgmj.64.753.559 3074289 PMC2428896

[B14] Barnden L. R. Shan Z. Y. Staines D. R. Marshall-Gradisnik S. Finegan K. Ireland T. (2019). Intra brainstem connectivity is impaired in chronic fatigue syndrome. *NeuroImage Clin.* 24:102045. 10.1016/j.nicl.2019.102045 31671321 PMC6835065

[B15] Beerlage C. Varanat M. Linder K. Maggi R. G. Cooley J. Kempf V. A. J. (2012). Bartonella vinsonii subsp. berkhoffii and Bartonella henselae as potential causes of proliferative vascular diseases in animals. *Med. Microbiol. Immunol.* 201 319–326. 10.1007/s00430-012-0234-5 22450733

[B16] Belay E. D. Abrams J. Oster M. E. Giovanni J. Pierce T. Meng L. (2021). Trends in geographic and temporal distribution of US children with multisystem inflammatory syndrome during the Covid-19 pandemic. *JAMA Pediatr.* 10.1001/jamapediatrics.20210630 Online ahead of print. PMC8025123 33821923

[B17] Belizário J. E. Faintuch J. (2018). Microbiome and gut dysbiosis. *Exp. Suppl.* 109 459–476. 10.1007/978-3-319-74932-7_13 30535609

[B18] Berlit P. Bösel J. Gahn G. Isenmann S. Meuth S. G. Nolte C. H. (2020). “Neurological manifestations of COVID-19” - guideline of the German society of neurology. *Neurol. Res. Pract.* 2:51. 10.1186/s42466-020-00097-7 33283160 PMC7708894

[B19] Bertina R. M. (1997). Factor V Leiden and other coagulation factor mutations affecting thrombotic risk. *Clin. Chem.* 43 1678–1683. 10.1093/clinchem/43.9.1678 9299960

[B20] Bose R. J. C. McCarthy J. R. (2020). Direct SARS-CoV-2 infection of the heart potentiates the cardiovascular sequelae of COVID-19. *Drug Discov. Today* 25 1559–1560. 10.1016/j.drudis.2020.06.021 32592868 PMC7313487

[B21] Bowie A. G. Unterholzner L. (2008). Viral evasion and subversion of pattern-recognition receptor signalling. *Nat. Rev. Immunol.* 8 911–922. 10.1038/nri2436 18989317 PMC7097711

[B22] Bragée B. Michos A. Drum B. Fahlgren M. Szulkin R. Bertilson B. C. (2020). Signs of intracranial hypertension, hypermobility, and craniocervical obstructions in patients with myalgic encephalomyelitis/chronic fatigue syndrome. *Front. Neurol.* 11:828. 10.3389/fneur.2020.00828 PMC7485557 32982905

[B23] Brinker T. Stopa E. Morrison J. Klinge P. (2014). A new look at cerebrospinal fluid circulation. *Fluids Barriers CNS* 11:10. 10.1186/2045-8118-11-10 24817998 PMC4016637

[B24] Brodin P. Jojic V. Gao T. Bhattacharya S. Angel C. J. L. Furman D. (2015). Variation in the human immune system is largely driven by non-heritable influences. *Cell* 160 37–47. 10.1016/j.cell.2014.12.020 25594173 PMC4302727

[B25] Cao X. (2020). COVID-19: immunopathology and its implications for therapy. *Nat. Rev. Immunol.* 20 269–270. 10.1038/s41577-020-0308-3 32273594 PMC7143200

[B26] Carding S. Verbeke K. Vipond D. T. Corfe B. M. Owen L. J. (2015). Dysbiosis of the gut microbiota in disease. *Microb. Ecol. Health Dis.* 2015:26. 10.3402/mehd.v26.26191 PMC4315779 25651997

[B27] Carfì A. Bernabei R. Landi F. (2020). Persistent symptoms in patients after acute COVID-19. *J. Am. Med. Assoc.* 324 603–605. 10.1001/jama.2020.12603 32644129 PMC7349096

[B28] Carruthers B. M. Van de Sande M. I. De Meirleir K. L. Klimas N. G. Broderick G. Mitchell T. (2011). Myalgic encephalomyelitis: international consensus criteria. *J. Intern. Med.* 270 327–338. 21777306 10.1111/j.1365-2796.2011.02428.x PMC3427890

[B29] Castro I. A. Jorge D. M. M. Ferreri L. M. Martins R. B. Pontelli M. C. Jesus B. L. S. (2020). Silent infection of B and CD8 + T lymphocytes by influenza a virus in children with tonsillar hypertrophy. *J. Virol.* 94:e01969-19. 10.1128/JVI.01969-19 PMC7163139 32075928

[B30] CDC (2019). *CDC - Toxoplasmosis - Epidemiology & Risk Factors.* Atlanta, GA: CDC.

[B31] Chan P. K. S. To K. F. Lo A. W. I. Cheung J. L. K. Chu I. Au F. W. L. (2004). Persistent infection of SARS coronavirus in colonic cells in vitro. *J. Med. Virol.* 74 1–7. 10.1002/jmv.20138 15258961 PMC7166317

[B32] Chen L. Y. C. Quach T. T. T. (2021). COVID-19 cytokine storm syndrome: a threshold concept. *Lancet Microbe* 2 e49–e50. 33655230 10.1016/S2666-5247(20)30223-8 PMC7906728

[B33] Chen S. H. Garber D. A. Schaffer P. A. Knipe D. M. Coen D. M. (2000). Persistent elevated expression of cytokine transcripts in ganglia latently infected with herpes simplex virus in the absence of ganglionic replication or reactivation. *Virology* 278 207–216. 10.1006/viro.2000.0643 11112495

[B34] Chen T. Song J. Liu H. Zheng H. Chen C. (2020). Positive epstein-barr virus detection in corona virus disease 2019 (COVID-19) patients. *SSRN Electron. J.* 11:10902. 10.1038/s41598-021-90351-y PMC8149409 34035353

[B35] Cheng H. Y. Ning M. X. Chen D. K. Ma W. T. (2019). Interactions between the gut microbiota and the host innate immune response against pathogens. *Front. Immunol.* 10:607. 10.3389/fimmu.2019.00607 PMC6449424 30984184

[B36] Chia J. K. S. Chia A. Y. (2008). Chronic fatigue syndrome is associated with chronic enterovirus infection of the stomach. *J. Clin. Pathol.* 61 43–48. 10.1136/jcp.2007.050054 17872383

[B37] Chia J. K. Chia A. Y. Wang D. El-Habbal R. (2015). Functional Dyspepsia and Chronic Gastritis Associated with Enteroviruses. *Open J. Gastroenterol.* 5 21–27. 10.4236/ojgas.2015.54005

[B38] Chia J. Chia A. Voeller M. Lee T. Chang R. (2010). Acute enterovirus infection followed by myalgic encephalomyelitis/chronic fatigue syndrome (ME/CFS) and viral persistence. *J. Clin. Pathol.* 63 165–168. 10.1136/jcp.2009.070466 19828908

[B39] Choi B. Choudhary M. C. Regan J. Sparks J. A. Padera R. F. Qiu X. (2020). Persistence and Evolution of SARS-CoV-2 in an immunocompromised host. *N. Engl. J. Med.* 383 2291–2293. 33176080 10.1056/NEJMc2031364 PMC7673303

[B40] Clark S. A. Clark L. E. Pan J. Coscia A. McKay L. G. A. Shankar S. (2021). SARS-CoV-2 evolution in an immunocompromised host reveals shared neutralization escape mechanisms. *Cell* 184 2605–2617.e18. 33831372 10.1016/j.cell.2021.03.027 PMC7962548

[B41] Clayton E. W. (2015). *Beyond Myalgic Encephalomyelitis/Chronic Fatigue Syndrome.* Washington, DC: National Academies Press.

[B42] Corman V. M. Muth D. Niemeyer D. Drosten C. (2018). Hosts and sources of endemic human coronaviruses. *Adv. Virus Res.* 100 163–188. 10.1016/bs.aivir.2018.01.001 29551135 PMC7112090

[B43] Cryan J. F. O’Riordan K. J. Sandhu K. Peterson V. Dinan T. G. (2020). The gut microbiome in neurological disorders. *Lancet Neurol.* 19 179–194. 31753762 10.1016/S1474-4422(19)30356-4

[B44] Cunningham J. W. Vaduganathan M. Claggett B. L. Jering K. S. Bhatt A. S. Rosenthal N. (2021). Clinical outcomes in young US adults hospitalized with COVID-19. *JAMA Intern. Med.* 181 379–381. 10.1001/jamainternmed.2020.5313 32902580 PMC7489373

[B45] Cunningham L. Bowles N. E. Archard L. C. (1991). Persistent virus infection of muscle in postviral fatigue syndrome. *Br. Med. Bull.* 47 852–871. 10.1093/oxfordjournals.bmb.a072516 1665379

[B46] Cusick M. F. Libbey J. E. Fujinami R. S. (2012). Molecular mimicry as a mechanism of autoimmune disease. *Clin. Rev. Allergy Immunol.* 42 102–111. 22095454 10.1007/s12016-011-8294-7 PMC3266166

[B47] Dando S. J. Mackay-Sim A. Norton R. Currie B. J. St. John J. A. Ekberg J. A. K. (2014). Pathogens penetrating the central nervous system: infection pathways and the cellular and molecular mechanisms of invasion. *Clin. Microbiol. Rev.* 27 691–726. 10.1128/cmr.00118-13 25278572 PMC4187632

[B48] Darbeheshti F. Mahdiannasser M. Uhal B. D. Ogino S. Gupta S. Rezaei N. (2021). Interindividual immunogenic variants: susceptibility to coronavirus, respiratory syncytial virus and influenza virus. *Rev. Med. Virol.* 10.1002/rmv.2234 Online ahead of print. 33724604 PMC8250219

[B49] Davalos D. Kyu Ryu J. Merlini M. Baeten K. M. Le Moan N. Petersen M. A. (2012). Fibrinogen-induced perivascular microglial clustering is required for the development of axonal damage in neuroinflammation. *Nat. Commun.* 3:1227. 10.1038/ncomms2230 PMC3514498 23187627

[B50] Davis H. E. Assaf G. S. McCorkell L. Wei H. Low R. J. Re’em Y. (2020). Characterizing long COVID in an international cohort: 7 months of symptoms and their impact. *medRxiv* [Preprint] 10.1101/2020.12.24.20248802 PMC8280690 34308300

[B51] Davis M. M. (2015). Flexibility for specificity. *Science* 347 371–372. 10.1126/science.aaa5082 25613876

[B52] de Melo G. D. Lazarini F. Levallois S. Hautefort C. Michel V. Larrous F. (2020). COVID-19-associated olfactory dysfunction reveals SARS-CoV-2 neuroinvasion and persistence in the olfactory system. *bioRxiv* [Preprint] 10.1101/2020.11.18.388819

[B53] Decman V. Kinchington P. R. Harvey S. A. K. Hendricks R. L. (2005). Gamma interferon can block herpes simplex virus type 1 reactivation from latency, even in the presence of late gene expression. *J. Virol.* 79 10339–10347. 10.1128/jvi.79.16.10339-10347.2005 16051826 PMC1182646

[B54] Del Rio C. Collins L. F. Malani P. (2020). Long-term health consequences of COVID-19. *J. Am. Med. Assoc.* 324 1723–1724. 10.1001/jama.2020.19719 33031513 PMC8019677

[B55] Diao B. Wang C. Tan Y. Chen X. Liu Y. Ning L. (2020). Reduction and functional exhaustion of T cells in patients with coronavirus disease 2019 (COVID-19). *Front. Immunol.* 11:827. 10.3389/fimmu.2020.00827 PMC7205903 32425950

[B56] Doi T. Kwon H. J. Honda T. Sato H. Yoneda M. Kai C. (2016). Measles virus induces persistent infection by autoregulation of viral replication. *Sci. Rep.* 6:37163. 10.1038/srep37163 PMC5121633 27883010

[B57] Dolei A. Ibba G. Piu C. Serra C. (2019). Expression of HERV genes as possible biomarker and target in neurodegenerative diseases. *Int. J. Mol. Sci.* 20:3706. 10.3390/ijms20153706 31362360 PMC6696274

[B58] Doobay M. F. Talman L. S. Obr T. D. Tian X. Davisson R. L. Lazartigues E. (2007). Differential expression of neuronal ACE2 in transgenic mice with overexpression of the brain renin-angiotensin system. *Am. J. Physiol. Regul. Integr. Comp. Physiol.* 292 R373–R381. 16946085 10.1152/ajpregu.00292.2006 PMC1761128

[B59] Dowsett E. G. Ramsay A. M. McCartney R. A. Bell E. J. (1990). Myalgic encephalomyelitis a persistent enteroviral infection? *Postgrad. Med. J.* 66 526–530. 10.1136/pgmj.66.777.526 2170962 PMC2429637

[B60] Doxey A. C. McConkey B. J. (2013). Prediction of molecular mimicry candidates in human pathogenic bacteria. *Virulence* 4 453–466. 10.4161/viru.25180 23715053 PMC5359739

[B61] Drago F. Ciccarese G. Rebora A. Parodi A. (2021). Human herpesvirus-6, -7, and epstein-barr virus reactivation in pityriasis rosea during COVID-19. *J. Med. Virol.* 93 1850–1851. 10.1002/jmv.26549 32970319 PMC7537064

[B62] Eastment M. C. McClelland R. S. (2018). Vaginal microbiota and susceptibility to HIV. *AIDS* 32 687–698. 10.1097/qad.0000000000001768 29424773 PMC5957511

[B63] Escoll P. Buchrieser C. (2018). Metabolic reprogramming of host cells upon bacterial infection: why shift to a Warburg-like metabolism? *FEBS J.* 285 2146–2160. 10.1111/febs.14446 29603622

[B64] Escoll P. Platon L. Buchrieser C. (2019). Roles of mitochondrial respiratory complexes during infection. *Immunometabolism* 1:e190011.

[B65] Eslamifar Z. Behzadifard M. Soleimani M. Behzadifard S. (2020). Coagulation abnormalities in SARS-CoV-2 infection: overexpression tissue factor. *Thromb. J.* 18:38. 10.1186/s12959-020-00250-x PMC7737414 33323111

[B66] Esposito F. Angileri F. F. Kruse P. Cavallo L. M. Solari D. Esposito V. (2016). Fibrin sealants in dura sealing: a systematic literature review. *PLoS One* 11:e0151533. 10.1371/journal.pone.0151533 27119993 PMC4847933

[B67] Fabre A. L. Colotte M. Luis A. Tuffet S. Bonnet J. (2014). An efficient method for long-term room temperature storage of RNA. *Eur. J. Hum. Genet.* 22 379–385. 10.1038/ejhg.2013.145 23860045 PMC3925273

[B68] Fetissov S. O. Hamze Sinno M. Coëffier M. Bole-Feysot C. Ducrotté P. Hökfelt T. (2008). Autoantibodies against appetite-regulating peptide hormones and neuropeptides: putative modulation by gut microflora. *Nutrition* 24 348–359. 10.1016/j.nut.2007.12.006 18262391 PMC7126273

[B69] Gacek R. R. (2002). The biology of neurotropic viruses. *Adv. Otorhinolaryngol.* 60 1–11. 10.1159/000059259 12077894

[B70] Gaebler C. Wang Z. Lorenzi J. C. C. Muecksch F. Finkin S. Tokuyama M. (2021). Evolution of antibody immunity to SARS-CoV-2. *Nature* 591 639–644. 10.1038/s41586-021-03207-w 33461210 PMC8221082

[B71] Galland L. (2014). The gut microbiome and the brain. *J. Med. Food* 17 1261–1272. 25402818 10.1089/jmf.2014.7000 PMC4259177

[B72] García-Martínez F. J. Moreno-Artero E. Jahnke S. (2020). SARS-CoV-2 and EBV coinfection. *Med. Clin.* 155 319–320. 10.1016/j.medcle.2020.06.010 32953993 PMC7486856

[B73] Gebbia J. A. Coleman J. L. Benach J. L. (2001). Borrelia spirochetes upregulate release and activation of matrix metalloproteinase gelatinase B (MMP-9) and collagenase 1 (MMP-1) in human cells. *Infect. Immun.* 69 456–462. 10.1128/iai.69.1.456-462.2001 11119537 PMC97903

[B74] Genoni A. Canducci F. Rossi A. Broccolo F. Chumakov K. Bono G. (2017). Revealing enterovirus infection in chronic human disorders: an integrated diagnostic approach. *Sci. Rep.* 7:5013. 10.1038/s41598-017-04993-y PMC5504018 28694527

[B75] Ghoneim H. E. Thomas P. G. McCullers J. A. (2013). Depletion of alveolar macrophages during influenza infection facilitates bacterial superinfections. *J. Immunol.* 191 1250–1259. 10.4049/jimmunol.1300014 23804714 PMC4907362

[B76] Giloteaux L. Goodrich J. K. Walters W. A. Levine S. M. Ley R. E. Hanson M. R. (2016). Reduced diversity and altered composition of the gut microbiome in individuals with myalgic encephalomyelitis/chronic fatigue syndrome. *Microbiome* 4:30. 10.1186/s40168-016-0171-4 PMC4918027 27338587

[B77] Gow J. W. Behan W. M. H. Clements G. B. Woodall C. Riding M. Behan P. O. (1991). Enteroviral RNA sequences detected by polymerase chain reaction in muscle of patients with postviral fatigue syndrome. *Br. Med. J.* 302 692–696. 10.1136/bmj.302.6778.692 1850635 PMC1669122

[B78] Graham A. C. Temple R. M. Obar J. J. (2015). Mast cells and influenza a virus: association with allergic responses and beyond. *Front. Immunol.* 6:238. 10.3389/fimmu.2015.00238 PMC4435071 26042121

[B79] Grandi N. Tramontano E. (2018). Human endogenous retroviruses are ancient acquired elements still shaping innate immune responses. *Front. Immunol.* 9:2039. 10.3389/fimmu.2018.02039 PMC6139349 30250470

[B80] Grobler C. Maphumulo S. C. Grobbelaar L. M. Bredenkamp J. C. Laubscher G. J. Lourens P. J. (2020). Covid-19: the rollercoaster of fibrin(ogen), d-dimer, von willebrand factor, p-selectin and their interactions with endothelial cells, platelets and erythrocytes. *Int. J. Mol. Sci.* 21:5168. 10.3390/ijms21145168 32708334 PMC7403995

[B81] Gu L. Deng H. Ren Z. Zhao Y. Yu S. Guo Y. (2019). Dynamic changes in the microbiome and mucosal immune microenvironment of the lower respiratory tract by influenza virus infection. *Front. Microbiol.* 10:2491. 10.3389/fmicb.2019.02491 PMC6838016 31736922

[B82] Gubernatorova E. O. Gorshkova E. A. Polinova A. I. Drutskaya M. S. (2020). IL-6: relevance for immunopathology of SARS-CoV-2. *Cytokine Growth Factor Rev.* 53 13–24. 10.1016/j.cytogfr.2020.05.009 32475759 PMC7237916

[B83] Guler S. A. Ebner L. Beigelman C. Bridevaux P.-O. Brutsche M. Clarenbach C. (2021). Pulmonary function and radiological features four months after COVID-19: first results from the national prospective observational Swiss COVID-19 lung study. *Eur. Respir. J.* 57:2003690. 10.1183/13993003.03690-2020 33419891 PMC8082329

[B84] Gupta A. Madhavan M. V. Sehgal K. Nair N. Mahajan S. Sehrawat T. S. (2020). Extrapulmonary manifestations of COVID-19. *Nat. Med.* 26 1017–1032. 32651579 10.1038/s41591-020-0968-3 PMC11972613

[B85] Gupta S. K. Haigh B. J. Griffin F. J. Wheeler T. T. (2013). The mammalian secreted RNases: mechanisms of action in host defence. *Innate Immun.* 19 86–97. 10.1177/1753425912446955 22627784

[B86] Hamming I. Timens W. Bulthuis M. L. C. Lely A. T. Navis G. J. van Goor H. (2004). Tissue distribution of ACE2 protein, the functional receptor for SARS coronavirus. a first step in understanding SARS pathogenesis. *J. Pathol.* 203 631–637. 10.1002/path.1570 15141377 PMC7167720

[B87] Harapan B. N. Yoo H. J. (2021). Neurological symptoms, manifestations, and complications associated with severe acute respiratory syndrome coronavirus 2 (SARS-CoV-2) and coronavirus disease 19 (COVID-19). *J. Neurol.* 10.1007/s00415-021-10406-y Online ahead of print. 33486564 PMC7826147

[B88] Harley J. B. Chen X. Pujato M. Miller D. Maddox A. Forney C. (2018). Transcription factors operate across disease loci, with EBNA2 implicated in autoimmunity. *Nat. Genet.* 50 699–707. 10.1038/s41588-018-0102-3 29662164 PMC6022759

[B89] Hirsch A. J. Smith J. L. Haese N. N. Broeckel R. M. Parkins C. J. Kreklywich C. (2017). Zika Virus infection of rhesus macaques leads to viral persistence in multiple tissues. *PLoS Pathog.* 13:e1006219. 10.1371/journal.ppat.1006219 28278237 PMC5344528

[B90] Hiscott J. Alexandridi M. Muscolini M. Tassone E. Palermo E. Soultsioti M. (2020). The global impact of the coronavirus pandemic. *Cytokine Growth Factor Rev.* 53 1–9. 32487439 10.1016/j.cytogfr.2020.05.010 PMC7254014

[B91] Hoffmann M. Kleine-Weber H. Schroeder S. Krüger N. Herrler T. Erichsen S. (2020). SARS-CoV-2 cell entry depends on ACE2 and TMPRSS2 and is blocked by a clinically proven protease inhibitor. *Cell* 181 271–280.e8. 32142651 10.1016/j.cell.2020.02.052 PMC7102627

[B92] Horn C. C. Wallisch W. J. Homanics G. E. Williams J. P. (2014). Pathophysiological and neurochemical mechanisms of postoperative nausea and vomiting. *Eur. J. Pharmacol.* 722 55–66. 10.1016/j.ejphar.2013.10.037 24495419 PMC3915298

[B93] Hornef M. (2015). Pathogens, commensal symbionts, and pathobionts: discovery and functional effects on the host. *ILAR J.* 56 159–162. 10.1093/ilar/ilv007 26323625

[B94] Hottes A. K. Freddolino P. L. Khare A. Donnell Z. N. Liu J. C. Tavazoie S. (2013). Bacterial adaptation through loss of function. *PLoS Genet.* 9:e1003617. 10.1371/journal.pgen.1003617 23874220 PMC3708842

[B95] Houseley J. Tollervey D. (2009). The many pathways of RNA degradation. *Cell* 136 763–776. 10.1016/j.cell.2009.01.019 19239894

[B96] Hu Y. Kang Y. Liu X. Cheng M. Dong J. Sun L. (2020). Distinct lung microbial community states in patients with pulmonary tuberculosis. *Sci. China Life Sci.* 63 1522–1533. 10.1007/s11427-019-1614-0 32303963

[B97] Huang C. Huang L. Wang Y. Li X. Ren L. Gu X. (2021). 6-month consequences of COVID-19 in patients discharged from hospital: a cohort study. *Lancet* 397 220–232. 33428867 10.1016/S0140-6736(20)32656-8 PMC7833295

[B98] Huang C. Wang Y. Li X. Ren L. Zhao J. Hu Y. (2020). Clinical features of patients infected with 2019 novel coronavirus in Wuhan. China. *Lancet* 395 497–506. 31986264 10.1016/S0140-6736(20)30183-5 PMC7159299

[B99] Huang H. I. Shih S. R. (2015). Neurotropic enterovirus infections in the central nervous system. *Viruses* 7 6029–6044. 10.3390/v7112920 PMC4664993 26610549

[B100] Huang Y. Pinto M. D. Borelli J. L. Mehrabadi M. A. Abrihim H. Dutt N. (2021). COVID symptoms, symptom clusters, and predictors for becoming a long-hauler: looking for clarity in the haze of the pandemic. *medRxiv* [Preprint] 10.1101/2021.03.03.21252086 36154716 PMC9510954

[B101] Huits R. De Smet B. Ariën K. K. Van Esbroeck M. Bottieau E. Cnops L. (2017). Zika virus in semen: a prospective cohort study of symptomatic travellers returning to Belgium. *Bull. World Health Organ.* 95 802–809. 10.2471/blt.17.181370 29200521 PMC5710082

[B102] Ireland D. D. C. Manangeeswaran M. Lewkowicz A. P. Engel K. Clark S. M. Laniyan A. (2020). Long-term persistence of infectious Zika virus: inflammation and behavioral sequela in mice. *PLoS Pathog.* 16:e1008689. 10.1371/journal.ppat.1008689 33301527 PMC7728251

[B103] Kantonen J. Mahzabin S. Mäyränpää M. I. Tynninen O. Paetau A. Andersson N. (2020). Neuropathologic features of four autopsied COVID-19 patients. *Brain Pathol.* 30 1012–1016. 10.1111/bpa.12889 32762083 PMC7436498

[B104] Karuppan M. K. M. Devadoss D. Nair M. Chand H. S. Lakshmana M. K. (2021). SARS-CoV-2 infection in the central and peripheral nervous system-associated morbidities and their potential mechanism. *Mol. Neurobiol.* 58 2465–2480. 10.1007/s12035-020-02245-1 33439437 PMC7805264

[B105] Katze M. G. He Y. Gale M. (2002). Viruses and interferon: a fight for supremacy. *Nat. Rev. Immunol.* 2 675–687. 10.1038/nri888 12209136

[B106] Kedor C. Freitag H. Meyer-Arndt L.-A. Wittke K. Zoller T. Steinbeis F. (2021). Chronic COVID-19 syndrome and chronic fatigue syndrome (ME/CFS) following the first pandemic wave in Germany: a first analysis of a prospective observational study. *medRxiv* [Preprint]. Available online at: https://www.medrxiv.org/content/10.1101/2021.02.06.21249256v1 (accessed April 19, 2021).

[B107] Keita A. K. Vidal N. Toure A. Kalifa Diallo M. S. N’Fally M. Baize S. (2019). A 40-month follow-up of Ebola virus disease survivors in Guinea (Postebogui) reveals long-term detection of Ebola viral ribonucleic acid in semen and breast milk. *Open Forum Infect. Dis.* 6:ofz482. 10.1093/ofid/ofz482 PMC7047953 32128327

[B108] Kemp S. A. Collier D. A. Datir R. P. Ferreira I. A. T. M. Gayed S. Jahun A. (2021). SARS-CoV-2 evolution during treatment of chronic infection. *Nature* 592 277–282. 33545711 10.1038/s41586-021-03291-y PMC7610568

[B109] Kempf V. A. J. Lebiedziejewski M. Alitalo K. Wälzlein J. H. Ehehalt U. Fiebig J. (2005). Activation of hypoxia-inducible factor-1 in bacillary angiomatosis: evidence for a role of hypoxia-inducible factor-1 in bacterial infections. *Circulation* 111 1054–1062. 10.1161/01.cir.0000155608.07691.b7 15723970

[B110] Kenny B. J. Bordoni B. (2019). *Neuroanatomy, Cranial Nerve 10 (Vagus Nerve).* Treasure Island, FL: StatPearls Publishing 30725856

[B111] Khatiwada S. Subedi A. (2020). Lung microbiome and coronavirus disease 2019 (COVID-19): possible link and implications. *Hum. Microb. J.* 17:100073. 10.1016/j.humic.2020.100073 32835135 PMC7405772

[B112] Kim K.-S. Tracy S. Tapprich W. Bailey J. Lee C.-K. Kim K. (2005). 5′-terminal deletions occur in Coxsackievirus B3 during replication in murine hearts and cardiac myocyte cultures and correlate with encapsidation of negative-strand viral RNA. *J. Virol.* 79 7024–7041. 10.1128/jvi.79.11.7024-7041.2005 15890942 PMC1112132

[B113] Kitsou K. Kotanidou A. Paraskevis D. Karamitros T. Katzourakis A. Tedder R. (2020). Upregulation of human endogenous retroviruses in bronchoalveolar lavage fluid of COVID-19 patients. *medRxiv [preprint]* 10.1101/2020.05.10.20096958 PMC8510252 34612698

[B114] Kobayashi N. Oka N. Takahashi M. Shimada K. Ishii A. Tatebayashi Y. (2020). Human herpesvirus 6B greatly increases risk of depression by activating hypothalamic-pituitary -adrenal axis during latent phase of infection. *iScience* 23:101187. 10.1016/j.isci.2020.101187 32534440 PMC7298549

[B115] Komaroff A. L. (2006). Is human herpesvirus-6 a trigger for chronic fatigue syndrome? *J. Clin. Virol.* 37 S39–S46. 17276367 10.1016/S1386-6532(06)70010-5

[B116] Komaroff A. L. Bateman L. (2021). Will COVID-19 lead to myalgic encephalomyelitis/chronic fatigue syndrome? *Front. Med.* 7:606824. 10.3389/fmed.2020.606824 PMC7848220 33537329

[B117] Kowarsky M. Camunas-Soler J. Kertesz M. De Vlaminck I. Koh W. Pan W. (2017). Numerous uncharacterized and highly divergent microbes which colonize humans are revealed by circulating cell-free DNA. *Proc. Natl. Acad. Sci. U. S. A.* 114 9623–9628. 10.1073/pnas.1707009114 28830999 PMC5594678

[B118] Kraus R. J. Yu X. Cordes B. L. A. Sathiamoorthi S. Iempridee T. Nawandar D. M. (2017). Hypoxia-inducible factor-1α plays roles in Epstein-Barr virus’s natural life cycle and tumorigenesis by inducing lytic infection through direct binding to the immediate-early BZLF1 gene promoter. *PLoS Pathog.* 13:e1006404. 10.1371/journal.ppat.1006404 28617871 PMC5487075

[B119] Kraynak T. E. Marsland A. L. Wager T. D. Gianaros P. J. (2018). Functional neuroanatomy of peripheral inflammatory physiology: a meta-analysis of human neuroimaging studies. *Neurosci. Biobehav. Rev.* 94 76–92. 10.1016/j.neubiorev.2018.07.013 30067939 PMC6363360

[B120] Kreye J. Reincke S. M. Kornau H. C. Sánchez-Sendin E. Corman V. M. Liu H. (2020). A Therapeutic Non-self-reactive SARS-CoV-2 antibody protects from lung pathology in a COVID-19 hamster model. *Cell* 183 1058–1069.e19. 33058755 10.1016/j.cell.2020.09.049 PMC7510528

[B121] Krismer B. Weidenmaier C. Zipperer A. Peschel A. (2017). The commensal lifestyle of Staphylococcus aureus and its interactions with the nasal microbiota. *Nat. Rev. Microbiol.* 15 675–687. 10.1038/nrmicro.2017.104 29021598

[B122] Kristensson K. Norrby E. (1986). Persistence of RNA viruses in the central nervous system. *Annu. Rev. Microbiol.* 40 159–184. 10.1146/annurev.mi.40.100186.001111 3535644

[B123] Kumata R. Ito J. Takahashi K. Suzuki T. Sato K. (2020). A tissue level atlas of the healthy human virome. *BMC Biol.* 18:55. 10.1186/s12915-020-00785-5 PMC7269688 32493363

[B124] Kusalik A. Bickis M. Lewis C. Li Y. Lucchese G. Marincola F. M. (2007). Widespread and ample peptide overlapping between HCV and *Homo sapiens* proteomes. *Peptides* 28 1260–1267. 10.1016/j.peptides.2007.04.001 17485143

[B125] Lam M. H. B. Wing Y. K. Yu M. W. M. Leung C. M. Ma R. C. W. Kong A. P. S. (2009). Mental morbidities and chronic fatigue in severe acute respiratory syndrome survivors long-term follow-up. *Arch. Intern. Med.* 169 2142–2147. 10.1001/archinternmed.2009.384 20008700

[B126] Lancman G. Mascarenhas J. Bar-Natan M. (2020). Severe COVID-19 virus reactivation following treatment for B cell acute lymphoblastic leukemia. *J. Hematol. Oncol.* 13:131. 10.1186/s13045-020-00968-1 PMC7531062 33008453

[B127] Larsen J. R. Martin M. R. Martin J. D. Kuhn P. Hicks J. B. (2020). Modeling the onset of symptoms of COVID-19. *Front. Public Health* 8:473. 10.3389/fpubh.2020.00473 PMC7438535 32903584

[B128] Lauer A. N. Tenenbaum T. Schroten H. Schwerk C. (2018). The diverse cellular responses of the choroid plexus during infection of the central nervous system. *Am. J. Physiol. Cell Physiol.* 314 C152–C165. 29070490 10.1152/ajpcell.00137.2017

[B129] Lee M.-H. Perl D. P. Nair G. Li W. Maric D. Murray H. (2021). Microvascular injury in the brains of patients with Covid-19. *N. Engl. J. Med.* 384 481–483. 10.1056/nejmc2033369 33378608 PMC7787217

[B130] Leibovitch E. C. Caruso B. Ha S. K. Schindler M. K. Lee N. J. Luciano N. J. (2018). Herpesvirus trigger accelerates neuroinflammation in a nonhuman primate model of multiple sclerosis. *Proc. Natl. Acad. Sci. U. S. A.* 115 11292–11297. 10.1073/pnas.1811974115 30322946 PMC6217390

[B131] Le-Trilling V. T. K. Trilling M. (2015). Attack, parry and riposte: molecular fencing between the innate immune system and human herpesviruses. *Tissue Antigens* 86 1–13. 10.1111/tan.12594 26061653

[B132] Lewis J. M. Clifford S. Nsutebu E. (2015). Toxoplasmosis in immunosuppressed patients. *Rheumatology* 54 1939–1940. 10.1093/rheumatology/kev115 25969518

[B133] Li Y. Masaki T. Yamane D. McGivern D. R. Lemon S. M. (2013). Competing and noncompeting activities of miR-122 and the 5′ exonuclease Xrn1 in regulation of hepatitis C virus replication. *Proc. Natl. Acad. Sci. U. S. A.* 110 1881–1886. 10.1073/pnas.1213515110 23248316 PMC3562843

[B134] Lima M. Siokas V. Aloizou A. M. Liampas I. Mentis A. F. A. Tsouris Z. (2020). Unraveling the possible routes of SARS-COV-2 invasion into the central nervous system. *Curr. Treat. Options Neurol.* 22:37. 10.1007/s11940-020-00647-z PMC7515807 32994698

[B135] Liotti F. M. Menchinelli G. Marchetti S. Posteraro B. Landi F. Sanguinetti M. (2020). Assessment of SARS-CoV-2 RNA test results among patients who recovered from COVID-19 with prior negative results. *JAMA Intern. Med.* 181 702–704. 10.1001/jamainternmed.2020.7570 33180119 PMC7662488

[B136] Lo T. Q. Marston B. J. Dahl B. A. De Cock K. M. (2017). Ebola: anatomy of an epidemic. *Annu. Rev. Med.* 68 359–370. 27813879 10.1146/annurev-med-052915-015604

[B137] Logue J. K. Franko N. M. McCulloch D. J. McConald D. Magedson A. Wolf C. R. (2021). Sequelae in adults at 6 months after COVID-19 Infection. *JAMA Netw. Open* 4:210830. 10.1001/jamanetworkopen.2021.0830 PMC7896197 33606031

[B138] Lucas M. J. Brouwer M. C. Van Der Ende A. Van De Beek D. (2013). Endocarditis in adults with bacterial meningitis. *Circulation* 127 2056–2062. 10.1161/circulationaha.113.001545 23596007

[B139] Lucas M. Karrer U. Lucas A. Klenerman P. (2001). Viral escape mechanisms - escapology taught by viruses. *Int. J. Exp. Pathol.* 82 269–286. 10.1046/j.1365-2613.2001.00204.x 11703537 PMC2517780

[B140] Lukiw W. J. Pogue A. Hill J. M. (2020). SARS-CoV-2 infectivity and neurological targets in the brain. *Cell. Mol. Neurobiol.* 10.1007/s10571-020-00947-947 Epub ahead of print. PMC7445393 32840758

[B141] Luo G. G. Ou J. H. J. (2015). Oncogenic viruses and cancer. *Virol. Sin.* 30 83–84. 25924992 10.1007/s12250-015-3599-y PMC4731227

[B142] Madeira A. Burgelin I. Perron H. Curtin F. Lang A. B. Faucard R. (2016). MSRV envelope protein is a potent, endogenous and pathogenic agonist of human toll-like receptor 4: relevance of GNbAC1 in multiple sclerosis treatment. *J. Neuroimmunol.* 291 29–38. 10.1016/j.jneuroim.2015.12.006 26857492

[B143] Magnus P. Gunnes N. Tveito K. Bakken I. J. Ghaderi S. Stoltenberg C. (2015). Chronic fatigue syndrome/myalgic encephalomyelitis (CFS/ME) is associated with pandemic influenza infection, but not with an adjuvanted pandemic influenza vaccine. *Vaccine* 33 6173–6177. 10.1016/j.vaccine.2015.10.018 26475444

[B144] Mantia R. Di Gesù M. Vetro A. Mantia F. Palma S. Iovane A. (2015). Shortness of filum terminale represents an anatomical specific feature in fibromyalgia: a nuclear magnetic resonance and clinical study. *Muscles. Ligaments Tendons J.* 5 33–37. 10.32098/mltj.01.2015.07 25878985 PMC4396674

[B145] Marino Gammazza A. Légaré S. Lo Bosco G. Fucarino A. Angileri F. Oliveri M. (2021). Molecular mimicry in the post-COVID-19 signs and symptoms of neurovegetative disorders? *Lancet Microbe* 2:e94. 10.1016/s2666-5247(21)00033-1 35544159

[B146] Marouf N. Cai W. Said K. N. Daas H. Diab H. Chinta V. R. (2021). Association between periodontitis and severity of COVID-19 infection: a case–control study. *J. Clin. Periodontol.* 48 483–491. 10.1111/jcpe.13435 33527378 PMC8014679

[B147] Masato A. Plotegher N. Boassa D. Bubacco L. (2019). Impaired dopamine metabolism in Parkinson’s disease pathogenesis. *Mol. Neurodegener.* 14:35. 10.1186/s13024-019-0332-6 PMC6728988 31488222

[B148] Matschke J. Lütgehetmann M. Hagel C. Sperhake J. P. Schröder A. S. Edler C. (2020). Neuropathology of patients with COVID-19 in Germany: a post-mortem case series. *Lancet Neurol.* 19 919–929. 10.1016/s1474-4422(20)30308-2 33031735 PMC7535629

[B149] McCallum M. Bassi J. De Marco A. Chen A. Walls A. C. Di Iulio J. (2021). SARS-CoV-2 immune evasion by variant B.1.427/B.1.429. *bioRxiv* [Preprint]. Available online at: 10.1101/2021.03.31.437925 (accessed April 19, 2021).

[B150] McDonald L. T. (2021). Healing after COVID-19: are survivors at risk for pulmonary fibrosis? *Am. J. Physiol. Lung Cell. Mol. Physiol.* 320 L257–L265. 33355522 10.1152/ajplung.00238.2020 PMC7900916

[B151] McGarry F. Gow J. Behan P. O. (1994). Enterovirus in the chronic fatigue syndrome. *Ann. Intern. Med.* 120 972–973. 10.7326/0003-4819-120-11-199406010-00020 8172448

[B152] Mead P. S. Duggal N. K. Hook S. A. Delorey M. Fischer M. Olzenak (2018). Zika virus shedding in semen of symptomatic infected men. *N. Engl. J. Med.* 378 1377–1385. 10.1056/nejmoa1711038 29641964

[B153] Meinhardt J. Radke J. Dittmayer C. Franz J. Thomas C. Mothes R. (2021). Olfactory transmucosal SARS-CoV-2 invasion as a port of central nervous system entry in individuals with COVID-19. *Nat. Neurosci.* 24 168–175. 33257876 10.1038/s41593-020-00758-5

[B154] Meyer B. J. Schmaljohn C. S. (2000). Persistent hantavirus infections: characteristics and mechanisms. *Trends Microbiol.* 8 61–67. 10.1016/s0966-842x(99)01658-3 10664598

[B155] Meyer B. J. Southern P. J. (1997). A novel type of defective viral genome suggests a unique strategy to establish and maintain persistent lymphocytic choriomeningitis virus infections. *J. Virol.* 71 6757–6764. 10.1128/jvi.71.9.6757-6764.1997 9261400 PMC191956

[B156] Miller K. D. Matullo C. M. Milora K. A. Williams R. M. O’Regan K. J. Rall G. F. (2019). Immune-mediated control of a dormant neurotropic RNA virus infection. *J. Virol.* 93:e00241-19. 10.1128/JVI.00241-19 PMC6714802 31270232

[B157] Miskinyte M. Sousa A. Ramiro R. S. de Sousa J. A. M. Kotlinowski J. Caramalho I. (2013). The genetic basis of *Escherichia coli* pathoadaptation to macrophages. *PLoS Pathog.* 9:e1003802. 10.1371/journal.ppat.1003802 24348252 PMC3861542

[B158] Moffatt M. F. Cookson W. O. (2017). The lung microbiome in health and disease. *Clin. Med. Lond.* 17 525–529. 10.7861/clinmedicine.17-6-525 29196353 PMC6297685

[B159] Montalvan V. Lee J. Bueso T. De Toledo J. Rivas K. (2020). Neurological manifestations of COVID-19 and other coronavirus infections: a systematic review. *Clin. Neurol. Neurosurg*. 194:105921. 10.1016/j.clineuro.2020.105921 32422545 PMC7227498

[B160] Montoya J. G. Liesenfeld O. (2004). Toxoplasmosis. *Lancet* 363 1965–1976. 10.1016/S0140-6736(04)16412-X 15194258

[B161] Motozono C. Toyoda M. Zahradnik J. Ikeda T. Saito A. Tan T. S. (2021). An emerging SARS-CoV-2 mutant evading cellular immunity and increasing viral infectivity. *bioRxiv [preprint].* Available online at: https://www.biorxiv.org/content/10.1101/2021.04.02.438288v1 (accessed April 19, 2021).

[B162] Murray R. S. Brown B. Brain D. Cabirac G. F. (1992a). Detection of coronavirus RNA and antigen in multiple sclerosis brain. *Ann. Neurol.* 31 525–533. 10.1002/ana.410310511 1596089 PMC7159714

[B163] Murray R. S. Cai G. Y. Hoel K. Zhang J. Y. Soike K. F. Cabirac G. F. (1992b). Coronavirus infects and causes demyelination in primate central nervous system. *Virology* 188 274–284. 10.1016/0042-6822(92)90757-g 1314455 PMC7131451

[B164] Nakatomi Y. Mizuno K. Ishii A. Wada Y. Tanaka M. Tazawa S. (2014). Neuroinflammation in patients with chronic fatigue syndrome/myalgic encephalomyelitis: An11C-(R)-PK11195 PET study. *J. Nucl. Med.* 55 945–950. 10.2967/jnumed.113.131045 24665088

[B165] Nalbandian A. Sehgal K. Gupta A. Madhavan M. V. McGroder C. Stevens J. S. (2021). Post-acute COVID-19 syndrome. *Nat. Med*. 27 601–615. 10.1038/s41591-021-01283-z 33753937 PMC8893149

[B166] Neugent M. L. Hulyalkar N. V. Nguyen V. H. Zimmern P. E. De Nisco N. J. (2020). Advances in understanding the human urinary microbiome and its potential role in urinary tract infection. *MBio* 11 e218–e220. 10.1128/mBio.00218-20 PMC7188990 32345639

[B167] Newberry F. Hsieh S. Y. Wileman T. Carding S. R. (2018). Does the microbiome and virome contribute to myalgic encephalomyelitis/chronic fatigue syndrome? *Clin. Sci.* 132 523–542. 10.1042/cs20171330 29523751 PMC5843715

[B168] Ngô H. M. Zhou Y. Lorenzi H. Wang K. Kim T. K. Zhou Y. (2017). Toxoplasma modulates signature pathways of human epilepsy, neurodegeneration & cancer. *Sci. Rep.* 7:11496. 10.1038/s41598-017-10675-6 PMC5597608 28904337

[B169] Nicastri E. Castilletti C. Liuzzi G. Iannetta M. Capobianchi M. R. Ippolito G. (2016). Persistent detection of Zika virus RNA in semen for six months after symptom onset in a traveller returning from Haiti to Italy, February 2016. *Eurosurveillance* 21:30314. 10.2807/1560-7917.ES.2016.21.32.30314 PMC4998502 27541989

[B170] Oikarinen M. Tauriainen S. Oikarinen S. Honkanen T. Collin P. Rantala I. (2012). Type 1 diabetes is associated with enterovirus infection in gut mucosa. *Diabetes Metab. Res. Rev.* 61 687–691. 10.2337/db11-1157 22315304 PMC3282798

[B171] Olde Loohuis L. M. Mangul S. Ori A. P. S. Jospin G. Koslicki D. Yang H. T. (2018). Transcriptome analysis in whole blood reveals increased microbial diversity in schizophrenia. *Transl. Psychiatry* 8:96. 10.1038/s41398-018-0107-9 PMC5943399 29743478

[B172] Oran D. P. Topol E. J. (2020). Prevalence of asymptomatic SARS-CoV-2 infection: a narrative review. *Ann. Intern. Med.* 173 362–367. 32491919 10.7326/M20-3012 PMC7281624

[B173] Pablos J. L. Galindo M. Carmona L. Lledó A. Retuerto M. Blanco R. (2020). Clinical outcomes of hospitalised patients with COVID-19 and chronic inflammatory and autoimmune rheumatic diseases: a multicentric matched cohort study. *Ann. Rheum. Dis.* 79 1544–1549. 10.1136/annrheumdis-2020-218296 32796045

[B174] Palazon A. Goldrath A. W. Nizet V. Johnson R. S. (2014). HIF transcription factors, inflammation, and immunity. *Immunity* 41 518–528. 10.1016/j.immuni.2014.09.008 25367569 PMC4346319

[B175] Pardi N. Hogan M. J. Porter F. W. Weissman D. (2018). mRNA vaccines-a new era in vaccinology. *Nat. Rev. Drug Discov*. 17 261–279. 10.1038/nrd.2017.243 29326426 PMC5906799

[B176] Pellegrini L. Albecka A. Mallery D. L. Kellner M. J. Paul D. Carter A. P. (2020). SARS-CoV-2 infects the brain choroid plexus and disrupts the blood-CSF barrier in human brain organoids. *Cell Stem Cell* 27 951–961.e5. 33113348 10.1016/j.stem.2020.10.001 PMC7553118

[B177] Planas D. Bruel T. Grzelak L. Guivel-Benhassine F. Staropoli I. Porrot F. (2021). Sensitivity of infectious SARS-CoV-2 B.1.1.7 and B.1.351 variants to neutralizing antibodies. *Nat. Med.* 27 917–924. 33772244 10.1038/s41591-021-01318-5

[B178] Plebani M. (2021). Persistent viral RNA shedding in COVID-19: caution, not fear. *EBioMedicine* 64:103234. 10.1016/j.ebiom.2021.103234 33581642 PMC7873611

[B179] Poole B. D. Scofield R. H. Harley J. B. James J. A. (2006). Epstein-Barr virus and molecular mimicry in systemic lupus erythematosus. *Autoimmunity* 39 63–70. 10.1080/08916930500484849 16455583

[B180] Porzionato A. Macchi V. Parenti A. De Caro R. (2004). The distribution of mast cells in the human area postrema. *J. Anat.* 204 141–147. 10.1111/j.1469-7580.2004.00256.x 15032921 PMC1571242

[B181] Pretorius E. Venter C. Laubscher G. J. Lourens P. J. Steenkamp J. Kell D. B. (2020a). Prevalence of amyloid blood clots in COVID-19 plasma. *medRxiv* [Preprint] 10.1101/2020.07.28.20163543 PMC7670290 33203441

[B182] Pretorius E. Venter C. Laubscher G. J. Lourens P. J. Steenkamp J. Kell D. B. (2020b). Prevalence of readily detected amyloid blood clots in ‘unclotted’ Type 2 diabetes mellitus and COVID-19 plasma: a preliminary report. *Cardiovasc. Diabetol.* 19:193. 10.1186/s12933-020-01165-7 PMC7670290 33203441

[B183] Pretorius E. Vlok M. Venter C. Bezuidenhout J. A. Laubscher G. J. Steenkamp J. (2021). Persistent clotting protein pathology in Long COVID/post-acute sequelae of COVID-19 (PASC) is accompanied by increased levels of antiplasmin. *medRxiv* [Preprint]. Available online at: 10.1101/2021.05.21.21257578 (accessed April 19, 2021). PMC8381139 34425843

[B184] Proal A. D. Lindseth I. A. Marshall T. G. (2017). Microbe-microbe and host-microbe interactions drive microbiome dysbiosis and inflammatory processes. *Discov. Med.* 23 51–60. 28245427

[B185] Proal A. Marshall T. (2018). Myalgic encephalomyelitis/chronic fatigue syndrome in the era of the human microbiome: persistent pathogens drive chronic symptoms by interfering with host metabolism, gene expression, and immunity. *Front. Pediatr.* 6:373. 10.3389/fped.2018.00373 PMC6288442 30564562

[B186] Proal A. D. VanElzakker M. B. (2021). Pathogens hijack host cell metabolism: intracellular infection as a driver of the Warburg effect in cancer and other chronic inflammatory conditions. *Immunometabolism* 3:e210003. 10.20900/immunometab20210003

[B187] Puelles V. G. Lütgehetmann M. Lindenmeyer M. T. Sperhake J. P. Wong M. N. Allweiss L. (2020). Multiorgan and renal tropism of SARS-CoV-2. *N. Engl. J. Med.* 383 590–592. 32402155 10.1056/NEJMc2011400 PMC7240771

[B188] Puntmann V. O. Carerj M. L. Wieters I. Fahim M. Arendt C. Hoffmann J. (2020). Outcomes of cardiovascular magnetic resonance imaging in patients recently recovered from coronavirus disease 2019 (COVID-19). *JAMA Cardiol.* 5 1265–1273. 10.1001/jamacardio.2020.3557 32730619 PMC7385689

[B189] Qi F. Qian S. Zhang S. Zhang Z. (2020). Single cell RNA sequencing of 13 human tissues identify cell types and receptors of human coronaviruses. *Biochem. Biophys. Res. Commun.* 526 135–140. 10.1016/j.bbrc.2020.03.044 32199615 PMC7156119

[B190] Rai D. K. Sharma P. Kumar R. (2020). Post covid 19 pulmonary fibrosis- is it reversible? *Indian. J. Tuberc.* 10.1016/j.ijtb.2020.11.003 Epub ahead of print. 34099197 PMC7654356

[B191] Rajpal S. Tong M. S. Borchers J. Zareba K. M. Obarski T. P. Simonetti O. P. (2021). Cardiovascular magnetic resonance findings in competitive athletes recovering from COVID-19 infection. *JAMA Cardiol.* 6 116–118. 32915194 10.1001/jamacardio.2020.4916 PMC7489396

[B192] Ramírez-Labrada A. G. Isla D. Artal A. Arias M. Rezusta A. Pardo J. (2020). The influence of lung microbiota on lung carcinogenesis, immunity, and immunotherapy. *Trends Cancer* 6 86–97. 10.1016/j.trecan.2019.12.007 32061309

[B193] Randall R. E. Griffin D. E. (2017). Within host RNA virus persistence: mechanisms and consequences. *Curr. Opin. Virol.* 23 35–42. 10.1016/j.coviro.2017.03.001 28319790 PMC5474179

[B194] Rangon C.-M. Krantic S. Moyse E. Fougère B. (2020). The Vagal autonomic pathway of COVID-19 at the crossroad of Alzheimer’s disease and aging: a review of knowledge. *J. Alzheimer’s Dis. Rep.* 4 537–551. 10.3233/adr-200273 33532701 PMC7835993

[B195] Rasa S. Nora-Krukle Z. Henning N. Eliassen E. Shikova E. Harrer T. (2018). Chronic viral infections in myalgic encephalomyelitis/chronic fatigue syndrome (ME/CFS). *J. Transl. Med.* 16: 268. 10.1186/s12967-018-1644-y PMC6167797 30285773

[B196] Readhead B. Haure-Mirande J. V. Funk C. C. Richards M. A. Shannon P. Haroutunian V. (2018). Multiscale analysis of independent Alzheimer’s cohorts finds disruption of molecular, genetic, and clinical networks by human herpesvirus. *Neuron* 99 64–82.e7. 29937276 10.1016/j.neuron.2018.05.023 PMC6551233

[B197] Rebman A. W. Aucott J. N. (2020). Post-treatment lyme disease as a model for persistent symptoms in lyme disease. *Front. Med.* 7:57. 10.3389/fmed.2020.00057 PMC7052487 32161761

[B198] Ribero M. S. Jouvenet N. Dreux M. Nisole S. (2020). Interplay between SARS-CoV-2 and the type I interferon response. *PLoS Pathog.* 16:e1008737. 10.1371/journal.ppat.1008737 32726355 PMC7390284

[B199] Richardson J. (2001). Viral isolation from brain in myalgic encephalomyelitis. *J. Chronic Fatigue Syndr.* 9 15–19. 10.1300/j092v09n03_03

[B200] Riddell M. A. Moss W. J. Hauer D. Monze M. Griffin D. E. (2007). Slow clearance of measles virus RNA after acute infection. *J. Clin. Virol.* 39 312–317. 10.1016/j.jcv.2007.05.006 17625962

[B201] Robba C. Battaglini D. Pelosi P. Rocco P. R. M. (2020). Multiple organ dysfunction in SARS-CoV-2: MODS-CoV-2. *Expert Rev. Respir. Med.* 14 865–868. 10.1080/17476348.2020.1778470 32567404 PMC7441756

[B202] Robbins-Juarez S. Y. Qian L. King K. L. Stevens J. S. Husain S. A. Radhakrishnan J. (2020). Outcomes for patients with COVID-19 and acute kidney injury: a systematic review and meta-analysis. *Kidney Int. Rep.* 5 1149–1160. 10.1016/j.ekir.2020.06.013 32775814 PMC7314696

[B203] Rogers G. B. Keating D. J. Young R. L. Wong M. L. Licinio J. Wesselingh S. (2016). From gut dysbiosis to altered brain function and mental illness: mechanisms and pathways. *Mol. Psychiatry* 21 738–748. 10.1038/mp.2016.50 27090305 PMC4879184

[B204] Rohde S. (2012). “Inflammatory diseases of the meninges,” in *Inflammatory Diseases of the Brain*, ed. Hahnel S. (Berlin: Springer).

[B205] Rose N. R. (2016). Viral myocarditis. *Curr. Opin. Rheumatol.* 28 383–389. 27166925 10.1097/BOR.0000000000000303 PMC4948180

[B206] Rothhammer V. Borucki D. M. Tjon E. C. Takenaka M. C. Chao C. C. Ardura-Fabregat A. (2018). Microglial control of astrocytes in response to microbial metabolites. *Nature* 557 724–728. 10.1038/s41586-018-0119-x 29769726 PMC6422159

[B207] Rowe P. C. Marden C. L. Heinlein S. Edwards C. C. (2018). Improvement of severe myalgic encephalomyelitis/chronic fatigue syndrome symptoms following surgical treatment of cervical spinal stenosis. *J. Transl. Med.* 16:21. 10.1186/s12967-018-1397-7 PMC5796598 29391028

[B208] Rupprecht T. A. Koedel U. Fingerle V. Pfister H. W. (2008). The pathogenesis of lyme neuroborreliosis: from infection to inflammation. *Mol. Med.* 14 205–212. 10.2119/2007-00091.rupprecht 18097481 PMC2148032

[B209] Sahni S. K. Rydkina E. (2009). Host-cell interactions with pathogenic Rickettsia species. *Future Microbiol.* 4 323–339. 19327117 10.2217/FMB.09.6 PMC2775711

[B210] Scarpellini E. Ianiro G. Attili F. Bassanelli C. De Santis A. Gasbarrini A. (2015). The human gut microbiota and virome: potential therapeutic implications. *Dig. Liver Dis.* 47 1007–1012. 10.1016/j.dld.2015.07.008 26257129 PMC7185617

[B211] Sclocco R. Beissner F. Bianciardi M. Polimeni J. R. Napadow V. (2018). Challenges and opportunities for brainstem neuroimaging with ultrahigh field MRI. *Neuroimage* 168 412–426. 10.1016/j.neuroimage.2017.02.052 28232189 PMC5777900

[B212] Seigneurin J. M. Guilbert B. Bourgeat M. J. Avrameas S. (1988). Polyspecific natural antibodies and autoantibodies secreted by human lymphocytes immortalized with Epstein-Barr virus. *Blood* 71 581–585. 10.1182/blood.v71.3.581.581 2830925

[B213] Sellner J. Simon F. Meyding-Lamade U. Leib S. L. (2006). Herpes-simplex virus encephalitis is characterized by an early MMP-9 increase and collagen type IV degradation. *Brain Res.* 1125 155–162. 10.1016/j.brainres.2006.09.093 17109833

[B214] Sender R. Fuchs S. Milo R. (2016). Revised estimates for the number of human and bacteria cells in the body. *PLoS Biol.* 14:e1002533. 10.1371/journal.pbio.1002533 27541692 PMC4991899

[B215] Shan Z. Y. Barnden L. R. Kwiatek R. A. Bhuta S. Hermens D. F. Lagopoulos J. (2020). Neuroimaging characteristics of myalgic encephalomyelitis/chronic fatigue syndrome (ME/CFS): a systematic review. *J. Transl. Med.* 18:335. 10.1186/s12967-020-02506-6 PMC7466519 32873297

[B216] Shen Z. Xiao Y. Kang L. Ma W. Shi L. Zhang L. (2020). Genomic diversity of severe acute respiratory syndrome-coronavirus 2 in patients with coronavirus disease 2019. *Clin. Infect. Dis.* 71 713–720. 32129843 10.1093/cid/ciaa203 PMC7108196

[B217] Shin W. Kim H. J. (2018). Intestinal barrier dysfunction orchestrates the onset of inflammatory host-microbiome cross-talk in a human gut inflammation-on-a-chip. *Proc. Natl. Acad. Sci. U. S. A.* 115 E10539–E10547. 30348765 10.1073/pnas.1810819115 PMC6233106

[B218] Silver R. Curley J. P. (2013). Mast cells on the mind: new insights and opportunities. *Trends Neurosci.* 36 513–521. 10.1016/j.tins.2013.06.001 23845731

[B219] Singh B. Fleury C. Jalalvand F. Riesbeck K. (2012). Human pathogens utilize host extracellular matrix proteins laminin and collagen for adhesion and invasion of the host. *FEMS Microbiol. Rev.* 36 1122–1180. 10.1111/j.1574-6976.2012.00340.x 22537156

[B220] Solomon T. (2021). Neurological infection with SARS-CoV-2 — the story so far. *Nat. Rev. Neurol.* 17 65–66. 10.1038/s41582-020-00453-w 33414554 PMC7789883

[B221] Song E. Zhang C. Israelow B. Lu P. Weizman O. El (2020). Neuroinvasive potential of SARS-CoV-2 revealed in a human brain organoid model. *bioRxiv* [Preprint] 10.1101/2020.06.25.169946 32935108 PMC7491522

[B222] Song E. Zhang C. Israelow B. Lu-Culligan A. Prado A. V. Skriabine S. (2021). Neuroinvasion of SARS-CoV-2 in human and mouse brain. *J. Exp. Med.* 218:e20202135. 10.1084/jem.20202135 PMC7808299 33433624

[B223] Sorrentino S. (2010). The eight human “canonical” ribonucleases: molecular diversity, catalytic properties, and special biological actions of the enzyme proteins. *FEBS Lett.* 584 2194–2200. 10.1016/j.febslet.2010.04.018 20388512

[B224] Steed A. L. Christophi G. P. Kaiko G. E. Sun L. Goodwin V. M. Jain U. (2017). The microbial metabolite desaminotyrosine protects from influenza through type I interferon. *Science* 357 498–502. 10.1126/science.aam5336 28774928 PMC5753406

[B225] Steiner I. Kennedy P. G. Pachner A. R. (2007). The neurotropic herpes viruses: herpes simplex and varicella-zoster. *Lancet Neurol.* 6 1015–1028. 10.1016/s1474-4422(07)70267-3 17945155

[B226] Strandwitz P. (2018). Neurotransmitter modulation by the gut microbiota. *Brain Res.* 1693 128–133. 10.1016/j.brainres.2018.03.015 29903615 PMC6005194

[B227] Sun J. Xiao J. Sun R. Tang X. Liang C. Lin H. (2020). Prolonged persistence of SARS-CoV-2 RNA in body fluids. *Emerg. Infect. Dis.* 26 1834–1838. 10.3201/eid2608.201097 32383638 PMC7392422

[B228] Sylwester A. W. Mitchell B. L. Edgar J. B. Taormina C. Pelte C. Ruchti F. (2005). Broadly targeted human cytomegalovirus-specific CD4+ and CD8+ T cells dominate the memory compartments of exposed subjects. *J. Exp. Med.* 202 673–685. 10.1084/jem.20050882 16147978 PMC2212883

[B229] Tabacof L. Tosto-Mancuso J. Wood J. Cortes M. Kontorovich A. McCarthy D. (2020). Post-acute COVID-19 syndrome negatively impacts health and wellbeing despite less severe acute infection. *medRxiv* [Preprint] 10.1101/2020.11.04.20226126

[B230] Taefehshokr N. Taefehshokr S. Hemmat N. Heit B. (2020). Covid-19: perspectives on innate immune evasion. *Front. Immunol.* 11:580641. 10.3389/fimmu.2020.580641 PMC7554241 33101306

[B231] Talkington J. Nickell S. P. (1999). Borrelia burgdorferi spirochetes induce mast cell activation and cytokine release. *Infect. Immun.* 67 1107–1115. 10.1128/iai.67.3.1107-1115.1999 10024550 PMC96436

[B232] Tang J. W. Holmes C. W. (2017). Acute and chronic disease caused by enteroviruses. *Virulence* 8 1062–1065. 10.1080/21505594.2017.1308620 28362547 PMC5711444

[B233] Tauriainen S. Oikarinen S. Oikarinen M. Hyöty H. (2011). Enteroviruses in the pathogenesis of type 1 diabetes. *Semin. Immunopathol.* 33 45–55. 10.1007/s00281-010-0207-y 20424841

[B234] Tehrani H. A. Darnahal M. Nadji S. A. Haghighi S. (2021). COVID-19 re-infection or persistent infection in patient with acute myeloid leukaemia M3: a mini review. *New Microbes New Infect.* 39:100830. 10.1016/j.nmni.2020.100830 33425365 PMC7777517

[B235] Tetz G. Brown S. M. Hao Y. Tetz V. (2018). Parkinson’s disease and bacteriophages as its overlooked contributors. *Sci. Rep.* 8:10812. 10.1038/s41598-018-29173-4 PMC6050259 30018338

[B236] Thaker S. K. Ch’ng J. Christofk H. R. (2019). Viral hijacking of cellular metabolism. *BMC Biol.* 17:59. 10.1186/s12915-019-0678-9 PMC6637495 31319842

[B237] Thomas R. M. Jobin C. (2020). Microbiota in pancreatic health and disease: the next frontier in microbiome research. *Nat. Rev. Gastroenterol. Hepatol.* 17 53–64. 10.1038/s41575-019-0242-7 31811279

[B238] Thompson D. Sorenson J. Greenmyer J. Brissette C. A. Watt J. A. (2020). The Lyme disease bacterium, *Borrelia burgdorferi*, stimulates an inflammatory response in human choroid plexus epithelial cells. *PLoS One* 15:e0234993. 10.1371/journal.pone.0234993 32645014 PMC7347220

[B239] Tschöpe C. Ammirati E. Bozkurt B. Caforio A. L. P. Cooper L. T. Felix S. B. (2021). Myocarditis and inflammatory cardiomyopathy: current evidence and future directions. *Nat. Rev. Cardiol.* 18 169–193. 10.1038/s41569-020-00435-x 33046850 PMC7548534

[B240] Ueland T. Holter J. C. Holten A. R. Müller K. E. Lind A. Bekken G. K. (2020). Distinct and early increase in circulating MMP-9 in COVID-19 patients with respiratory failure: MMP-9 and respiratory failure in COVID-19. *J. Infect.* 81 e41–e43. 32603675 10.1016/j.jinf.2020.06.061 PMC7320854

[B241] Ursell L. K. Metcalf J. L. Parfrey L. W. Knight R. (2012). Defining the human microbiome. *Nutr. Rev.* 70 S38–S44. 22861806 10.1111/j.1753-4887.2012.00493.x PMC3426293

[B242] V’kovski P. Kratzel A. Steiner S. Stalder H. Thiel V. (2021). Coronavirus biology and replication: implications for SARS-CoV-2. *Nat. Rev. Microbiol.* 19 155–170. 10.1038/s41579-020-00468-6 33116300 PMC7592455

[B243] VanElzakker M. B. (2013). Chronic fatigue syndrome from vagus nerve infection: a psychoneuroimmunological hypothesis. *Med. Hypotheses* 81 414–423. 10.1016/j.mehy.2013.05.034 23790471

[B244] VanElzakker M. B. Brumfield S. A. Lara Mejia P. S. (2019). Neuroinflammation and cytokines in myalgic encephalomyelitis/chronic fatigue syndrome (ME/CFS): a critical review of research methods. *Front. Neurol.* 10:1033. 10.3389/fneur.2019.00316 PMC6454267 31001197

[B245] Vibholm L. K. Nielsen S. S. Pahus M. H. Frattari G. S. Olesen R. Andersen R. (2021). SARS-CoV-2 persistence is associated with antigen-specific CD8 T-cell responses. *EBioMedicine* 64:103230. 10.1016/j.ebiom.2021.103230 33530000 PMC7847186

[B246] Viola M. V. Scott C. Duffy P. D. (1978). Persistent measles virus infection in vitro and in man. *Arthritis Rheum.* 21 S46–S51. 78715

[B247] Virgin H. W. Wherry E. J. Ahmed R. (2009). Redefining chronic viral infection. *Cell* 138 30–50. 10.1016/j.cell.2009.06.036 19596234

[B248] Virological (2021). *Topics - Virological.* Available online at: https://virological.org/c/ebolavirus/guinea-2021/44 (accessed April 19, 2021).

[B249] Waldman B. S. Schwarz D. Wadsworth M. H. Saeij J. P. Shalek A. K. Lourido S. (2020). Identification of a master regulator of differentiation in toxoplasma. *Cell* 180 359–372.e16. 31955846 10.1016/j.cell.2019.12.013 PMC6978799

[B250] Wang E. Y. Mao T. Klein J. Dai Y. Huck J. D. Liu F. (2020). Diverse functional autoantibodies in patients with COVID-19. *medRxiv* [Preprint] 10.1101/2020.12.10.20247205 34010947 PMC13130511

[B251] Wang E. Y. Mao T. Klein J. Dai Y. Huck J. D. Jaycox J. R. (2021). Diverse functional autoantibodies in patients with COVID-19. *Nature* 10.1038/s41586-021-03631-y [Epub ahead of print]. 34010947 PMC13130511

[B252] Wang L. W. Shen H. Nobre L. Ersing I. Paulo J. A. Trudeau S. (2019). Epstein-barr-virus-induced one-carbon metabolism drives B cell transformation. *Cell Metab.* 30 539–555.e11. 31257153 10.1016/j.cmet.2019.06.003 PMC6720460

[B253] Wang T. T. Tavera-Mendoza L. E. Laperriere D. Libby E. MacLeod N. B. Nagai Y. (2005). Large-scale in Silico and microarray-based identification of direct 1,25-dihydroxyvitamin D3 target genes. *Mol. Endocrinol.* 19 2685–2695. 10.1210/me.2005-0106 16002434

[B254] Weber F. (2007). Interaction of hepatitis C virus with the type I interferon system. *World J. Gastroenterol.* 13 4818–4823. 10.3748/wjg.v13.i36.4818 17828812 PMC4611759

[B255] Werth N. Beerlage C. Rosenberger C. Yazdi A. S. Edelmann M. Amr A. (2010). Activation of hypoxia inducible factor 1 is a general phenomenon in infections with human pathogens. *PLoS One* 5:e0011576. 10.1371/journal.pone.0011576 PMC2904385 20644645

[B256] Wikoff W. R. Anfora A. T. Liu J. Schultz P. G. Lesley S. A. Peters E. C. (2009). Metabolomics analysis reveals large effects of gut microflora on mammalian blood metabolites. *Proc. Natl. Acad. Sci. U. S. A.* 106 3698–3703. 10.1073/pnas.0812874106 19234110 PMC2656143

[B257] Wilson H. W. Amo-Addae M. Kenu E. Ilesanmi O. S. Ameme D. K. Sackey S. O. (2018). Post-ebola syndrome among ebola virus disease survivors in montserrado county, Liberia 2016. *Biomed Res. Int.* 2018:1909410. 10.1155/2018/1909410 PMC6046154 30050920

[B258] Wing M. G. (1995). The molecular basis for a polyspecific antibody. *Clin. Exp. Immunol.* 99 313–315. 10.1111/j.1365-2249.1995.tb05551.x 7882551 PMC1534201

[B259] Wylie K. M. Mihindukulasuriya K. A. Zhou Y. Sodergren E. Storch G. A. Weinstock G. M. (2014). Metagenomic analysis of double-stranded DNA viruses in healthy adults. *BMC Med.* 12:71. 10.1186/s12915-014-0071-7 PMC4177058 25212266

[B260] Xiang Z. Koo H. Chen Q. Zhou X. Liu Y. Simon-Soro A. (2021). Potential implications of SARS-CoV-2 oral infection in the host microbiota. *J. Oral Microbiol.* 13:1853451. 10.1080/20002297.2020.1853451 33312449 PMC7711743

[B261] Xu H. Zhong L. Deng J. Peng J. Dan H. Zeng X. (2020). High expression of ACE2 receptor of 2019-nCoV on the epithelial cells of oral mucosa. *Int. J. Oral Sci.* 12:8. 10.1038/s41368-020-0074-x PMC7039956 32094336

[B262] Xu R. Zhou Y. Cai L. Wang L. Han J. Yang X. (2020). Co-reactivation of the human herpesvirus alpha subfamily (herpes simplex virus-1 and varicella zoster virus) in a critically ill patient with COVID-19. *Br. J. Dermatol.* 183 1145–1147. 10.1111/bjd.19484 32790074 PMC7436688

[B263] Yang B. Wei J. Ju P. Chen J. (2019). Effects of regulating intestinal microbiota on anxiety symptoms: a systematic review. *Gen. Psychiatry* 32:e100056. 10.1136/gpsych-2019-100056 31179435 PMC6551444

[B264] Yenamandra S. P. Hellman U. Kempkes B. Darekar S. D. Petermann S. Sculley T. (2010). Epstein-barr virus encoded EBNA-3 binds to vitamin D receptor and blocks activation of its target genes. *Cell. Mol. Life Sci.* 67 4249–4256. 10.1007/s00018-010-0441-4 20593215 PMC11115686

[B265] Yost S. Duran-Pinedo A. E. Teles R. Krishnan K. Frias-Lopez J. (2015). Functional signatures of oral dysbiosis during periodontitis progression revealed by microbial metatranscriptome analysis. *Genome Med.* 7:27. 10.1186/s13073-015-0153-3 PMC4410737 25918553

[B266] Yousef G. E. Mann G. F. Smith D. G. Bell E. J. Murugesan V. Mccartney R. A. (1988). Chronic enterovirus infection in patients with postviral fatigue syndrome. *Lancet* 331 146–150. 10.1016/s0140-6736(88)92722-5 2892990

[B267] Zhang X. Zheng Z. Shu B. Liu X. Zhang Z. Liu Y. (2013). Human astrocytic cells support persistent coxsackievirus B3 infection. *J. Virol.* 87 12407–12421. 10.1128/jvi.02090-13 24027313 PMC3807905

[B268] Zheng M. Gao Y. Wang G. Song G. Liu S. Sun D. (2020). Functional exhaustion of antiviral lymphocytes in COVID-19 patients. *Cell. Mol. Immunol.* 17 533–535. 10.1038/s41423-020-0402-2 32203188 PMC7091858

[B269] Zheng P. Bao L. Yang W. Wang J. J. (2020). Clinical symptoms between severe and non-severe COVID-19 pneumonia: a protocol for systematic review and meta-analysis. *Medicine.* 99:e21618. 10.1097/md.0000000000021618 32872018 PMC7437777

[B270] Zhu C. Zhu Q. Wang C. Zhang L. Wei F. Cai Q. (2016). Hostile takeover: manipulation of HIF-1 signaling in pathogen-associated cancers (Review). *Int. J. Oncol.* 49 1269–1276. 10.3892/ijo.2016.3633 27499495

[B271] Zhu Z. Lian X. Su X. Wu W. Marraro G. A. Zeng Y. (2020). From SARS and MERS to COVID-19: a brief summary and comparison of severe acute respiratory infections caused by three highly pathogenic human coronaviruses. *Respir. Res.* 21:224. 10.1186/s12931-020-01479-w PMC7450684 32854739

[B272] Zuo T. Zhang F. Lui G. C. Y. Yeoh Y. K. Li A. Y. L. Zhan H. (2020). Alterations in gut microbiota of patients With COVID-19 during time of hospitalization. *Gastroenterology* 159 944–955.e8. 32442562 10.1053/j.gastro.2020.05.048 PMC7237927

